# Evidence and possible mechanism of *Scutellaria baicalensis* and its bioactive compounds for hepatocellular carcinoma treatment

**DOI:** 10.1080/07853890.2023.2247004

**Published:** 2024-01-17

**Authors:** Ming-Yue Ma, Xiao-Ji Niu, Qian Wang, Shou-Mei Wang, Xin Li, Shu-Hui Zhang

**Affiliations:** a Department of Pathology, Yueyang Hospital of Integrated Traditional Chinese and Western Medicine, Shanghai University of Traditional Chinese Medicine, Shanghai, China; b Department of Dermatology, Yueyang Hospital of Integrated Traditional Chinese and Western Medicine, Shanghai University of Traditional Chinese Medicine, Shanghai, China; c Institute of Dermatology, Shanghai Academy of Traditional Chinese Medicine, Shanghai, China

**Keywords:** Scutellaria baicalensis, hepatocellular carcinoma, Baicalein (BAE;), Baicalin (BAI;), Wogonin (WOG)

## Abstract

**Background:**

Traditional Chinese medicines have been reported to have outstanding effects in the treating of hepatocellular carcinoma. *Scutellaria baicalensis* (*S. baicalensis*) has demonstrated anti-tumor, anti-angiogenic, and anti-inflammatory properties. Baicalein, wogonin, and baicalin are the main pharmacologically bioactive compounds of *S. baicalensis*.

**Methods:**

Eight electronic databases were searched to select articles published from their inception to 30 May 2022. For selected articles, clinical and preclinical data was obtained on the use of *S. baicalensis* and its bioactive compounds in hepatocellular carcinoma therapy. Statistical analyses were performed using RevMan version 5.3 and Stata software. Quality assessment of the studies was performed using Cochrane and Systematic Review Centre for Laboratory Animal Experimentation (SYRCLE)’s risk of bias tools.

**Results:**

Seven clinical and 17 preclinical *in vivo* studies along with 31 *in vitro* studies were included in this research. Meta-analysis showed that a Chinese herbal medicine preparation, with *S. baicalensis* as the sovereign herb, combined with Transcatheter arterial chemoembolization (TACE) or primary treatment, could lead to a significantly improved tumor objective response rate (Risk ratio (RR) = 1.57, 95% confidence interval (CI): [1.30, 1.90], *p* < 0.00001). *Scutellaria baicalensis*-based extracts (standard mean difference (SMD) = –0.86, 95%CI: [–1.20, −0.53], *p* < 0.00001), baicalein (SMD = –4.80, 95%CI: [–6.66, − 2.95], *p* < 0.00001), baicalin (SMD = –2.28, 95%CI [–3.26, −1.30], *p* < 0.00001) and wogonin (SMD = –1.41, 95%CI [–2.26, −0.57], *p* < 0.00001) slowed tumor growth *in vivo*. These outcomes might be linked to the mechanism by which *S. baicalensis* promotes apoptosis, induces autophagy, and blocks the expression of vascular endothelial growth factor (*p* < 0.05).

**Conclusion:**

Based on experimental and clinical evidence, we believe that *S. baicalensis* and its bioactive compounds have therapeutic potential and plausible mechanisms of action against hepatocellular carcinoma, in terms of efficacy and safety.

## 1. Introduction

**Figure 1.**
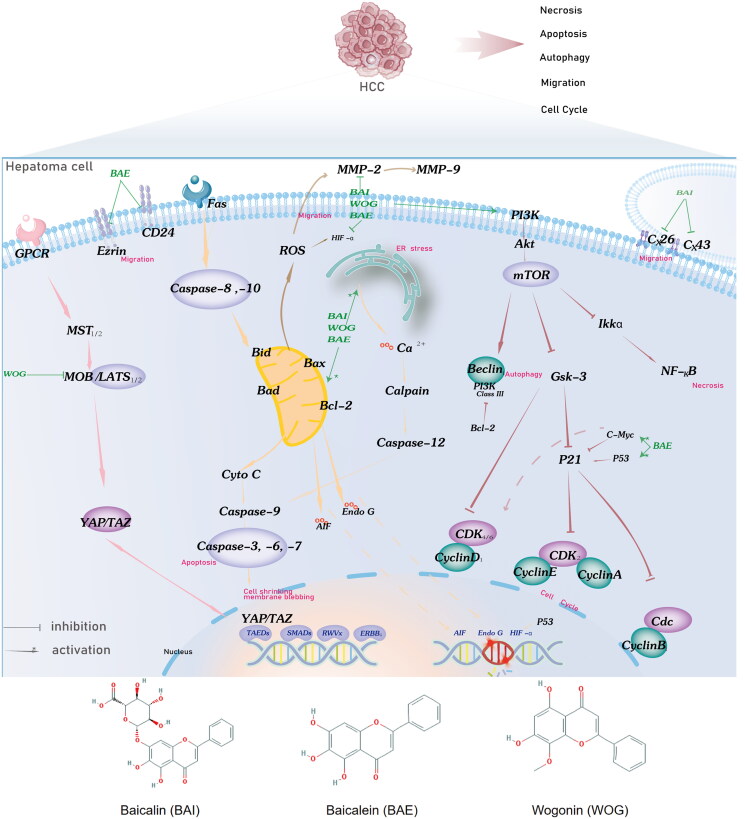
Potential BAI, BAE, and WOG action mechanisms in HCC treatment. BAE, BAI, and WOG affected cell apoptosis by inhibiting the expression of Bcl-2/Bax and migration by activating the ER stress and ROS pathway. BAE starts the effect of cell-cycle regulation by inhibiting the expression and function of cell-cycle genes, including CyclinE, CyclinD1, CyclinB1, CyclinA2, and CDK. BAE, BAI, and WOG induce autophagy, cell cycle, and necrosis by PI3K/Akt/mTOR, Hippo, and Nf-KB pathways. BAI and BAE inhibited migration by decreasing adhesion molecule release. Abbreviation: BAI, baicalin, BAE, baicalein, WOG, Wogonin.

Hepatocellular carcinoma (HCC) is the sixth most common carcinoma and the fourth leading cause of cancer-related deaths worldwide [1]. According to the GLOBOCAN Cancer Related database, the incidence of HCC is 18.2%, and the mortality rate is 17.2%, with more males than females in China being diagnosed [2]. In the next 20 years, the number of patients with HCC will stabilize at 100000–150000 based on epidemiology. In China, Hepatitis B virus infection is a major risk factor for HCC. Surgical resection and liver transplantation have always been regarded as the best radical treatments for HCC. Most patients have lost the opportunity for surgery due to poor conditions and limited clinical levels. A multidisciplinary team of national concern has been established to optimize the rationality of therapy [3]. Traditional Chinese medicine (CHM) is an important component of the multidisciplinary team for the treatment of liver cancer [4]. *Scutellaria baicalensis* (*S. baicalensis*; also called Huangqin) is a Chinese herb from the Labiatae family that is widely used in clinical adjuvant antitumor therapy [5,6]. It is a common cancer medication recorded in the Chinese Pharmacopoeia (2020) and European Pharmacopoeia (EP 9.0). The dried root section is used as a herbal medicine [7]. The main active components are flavonoids, saponins, and polysaccharides. Baicalein (BAE), baicalin (BAI), and wogonin (WOG) are all flavonoids that are major anticancer agents with excellent safety profiles [8–11]. These bioactive compounds have also been confirmed to possess cytostatic and cytotoxic activities against various human liver cancer cell lines. It is reassuring that these three flavonoids exhibit no (or very little) toxicity for normal epithelial cells, peripheral blood cells, leukocyte, and myeloid cells [8]. Their chemical structures are shown in Figure 1.

Several studies have reported that *S. baicalensis* plays an important role in the pharmacological activity of antitumors [12–14]. The mechanisms of *S. baicalensis* have also been demonstrated mainly *via* the apoptosis of hepatoma cells induced by mitochondria and cell death receptors, and with inhibition of cell proliferation. In addition to the above classical pathways, *S. baicalensis* can also induce autophagy of stem-cell receptors, regulate angiogenesis-related proteins and genes, and regulate the tumor immune microenvironment [15–18]. However, till date, there has been no systematic review on the efficacy and safety of *S. baicalensis* and its bioactive compounds in HCC therapy. Thus, we aimed to provide evidence for the potential mechanisms of action of these pathways. Here we present a comprehensive evaluation of the drug-herb interaction of *S. baicalensis* and its identified bioactive compounds from clinical and preclinical perspectives in the English and Chinese literature.

## 2. Methods

### 2.1. Protocol and registration

This review was conducted in accordance with the Preferred Reporting Items for Systematic Reviews and Meta-Analyses (PRISMA) guidelines [19]. The pre-designed review protocol was submitted to PROSPERO with the registration number (ID: CRD42022340270).

### 2.2. Data source

The selected articles were published from database inception to 30 May 2022. Data sources on the use of *S. baicalensis* and its bioactive compounds in HCC therapy were selected from the literature using eight electronic databases, including China BioMedical Bibliographic Database (www.imicams.ac.cn/cbm), TCMLARS (www.cintcm.com), PubMed (www.pubmed.gov), EMBASE (http://embase.com/), Cochrane Library (http://cochrane.org), CNKI (https://kns.cnki.net), Wanfang (https://www.wanfangdata.com.cn/index.html), and VIP (http://www.cqvip.com/). We combined MeSH subject headings and free words to develop the search formula. The search details and results are presented in Supplementary Table S1. The search strategy minimally involved all hepatocellular carcinoma, basic therapy, *S. baicalensis*, and its bioactive compounds, and randomized controlled trials (RCTs; all terms had associated synonyms). References from previously published analyses were collected as missing supplements.

### 2.3. Study selection

Clinical studies meeting the following criteria were considered eligible and included:(1) participants: humans; (2) patients clinically or pathologically diagnosed with liver cancer with typical imaging features (the age, sex, race, and severity of the disease were not restricted); (3) interventions that included the administration of CHM preparations with or without conventional treatment, including Transcatheter arterial chemoembolization (TACE), ablation, radiotherapy, and other basic therapies; and (4) CHM preparation with *S. baicalensis* as the sovereign herb. ‘Sovereign herb’ is the ingredient that provides a dominant curative action on the main disorder or primary symptoms, and has the greatest effect upon based on a standard published on 3 March 2022 WHO international standard terminologies on traditional Chinese medicine. This ingredient is indispensable to the formula. Based on the CONSORT Extension for Chinese Herbal Medicine Formulas 2017 [20], further details with formulation principles including sovereign herb were needed to be provided in the selection studies; (5) qualified research reported at least one of the following outcomes: tumor response, Karnofsky Performance Status (KPS) score, and immune and adverse events; and (6) RCTs.

For preclinical studies, the following criteria were used: (1) participants: experimental animals and cells; unlimited genus and species; (2) intervention drugs: *S. baicalensis*, BAE, BAI, and WOG; and (3) intervention trials acting on animal models and cell lines of hepatic cancer.

Studies confirming the following criteria were excluded:(1) studies and reviews not associated with *S. baicalensis* and its active composition; (2) reviews, experience, observational studies, duplicate articles, retrospective studies, and case reports; (3) no appropriate control group and predetermined efficacy indicators; and (4) raw data could not be applied by statistical processing.

### 2.4. Data extraction

Two researchers (M-YM and S-MW) independently screened titles and abstracts, applied the inclusion and exclusion criteria, and performed data extraction. When necessary, a third reviewer (S-HZ) was required to resolve any disagreement. Predesigned spreadsheets were used to record clinical data on patient characteristics, experimental and control details, clinical outcomes, and study quality [21]. For preclinical studies, we set up prefetch sheets to extract animal species, strain, male/female, age, type of transplantation, intervention composition, administration, and efficacy outcomes [22]. We corresponded with the author of the literature by requesting missing data *via* email.

### 2.5. Study quality

Two investigators (QW and X-JN) independently assessed the risk of bias for clinical research using the evaluation tool for RCTs recommended by the Cochrane Handbook for Systematic Reviews of Interventions, and the Systematic Review Centre for Laboratory Animal Experimentation (SYRCLE) risk-of-bias tool for preclinical research. The evaluation parameters of the risk-of-bias tool for RCTs were as follows: selection bias, blinding of implement bias, follow-up bias, measurement bias, reporting bias, and other biases. The SYRCLE tool was used to evaluate bias in six aspects: selection (sequence generation, baseline characteristics, and allocation concealment), performance (random housing and blinding), detection (random outcome assessment and blinding), attrition, reporting, and other performances. We assigned ‘low,’ ‘high’ or ‘unclear’ risk of bias to each article according to the level of evidence. To measure the differences attributable to clinical factors, methodology, and statistics, heterogeneity assessments using I^2^ statistics were performed by subgroup and sensitivity analyses [23,24].

### 2.6. Statistical analysis

All data for both continuous and categorical variables were analyzed using RevMan version 5.3. Statistical analysis for primary and secondary outcomes considered both a random-effects model and a fixed-effects model, in which the I^2^ statistic was used as adjustment evidence. We calculated the Risk Ratios (RR) and 95% confidence intervals (CIs) by collecting or converting the raw data of the trials. For continuous data, Standardized Mean Difference (SMD) was used because different measurement units existed.

### 2.7. Analysis of outcomes

The clinical outcomes for subsequent analyses were formulated according to the standard response evaluation criteria in solid tumors (RECIST). The primary outcome was tumor response, with a secondary outcome of the Karnofsky Performance Scale (KPS) and adverse events. For preclinical trials *in vivo*, we referred to Yuan et al. for the evaluation of efficacy and safety [25]. The primary outcome measure was the average weight of the transplanted animal tumors. The following indicators were used as secondary outcome measures: apoptosis of tumor cells, body weight after treatment, liver, kidney, spleen index, caspase-3, and vascular endothelial growth factor (VEGF). In preclinical trials *in vitro*, all cytokines in accordance with meta-outcomes were analyzed to clarify the mechanism of action of *S. baicalensis*, including Caspase-3, Bcl-2, MMP-2, MMP-9, and CyclinD1.

## 3. Results

### 3.1. Study selection

**Figure 2.**
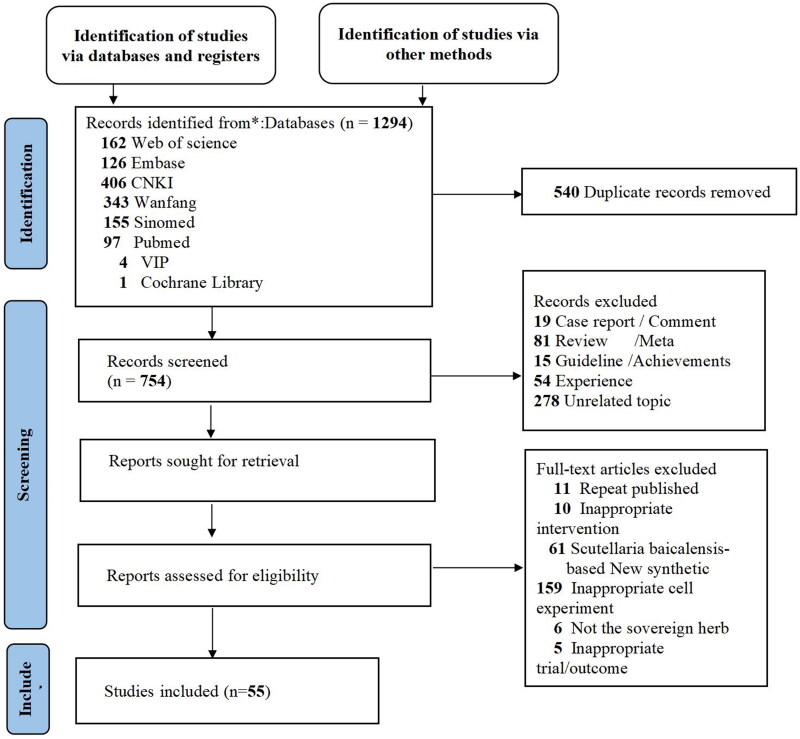
Literature search and study inclusion.

A total of 1294 articles were retrieved using a well-designed search strategy. In total, 540 duplicates were electronically and manually deleted. We screened 754 potential records on the basis of titles and abstracts and locked the full-text reading of 317 articles. The studies were rigorously reviewed according to the inclusion and exclusion criteria. There were six articles in which *S. baicalensis* was not the sovereign herb, 11 articles that were repeated publication, and 235 articles that did not meet the inclusion criteria for our study. Overall, seven clinical RCTs, 17 preclinical studies in laboratory animals, and 31 preclinical studies *in vitro* were reviewed. A PRISMA flow diagram is shown in Figure 2.

### 3.2. Characteristics of selection

#### 3.2.1. Clinical study

The main characteristics of the clinical studies are listed in Supplementary Table S2. The seven RCTs, ranging in sample size from 30 to 120, included 646 individuals, aged 18–72, with a definitive diagnosis of HCC. *Scutellaria. baicalensis* interventions in the CHM compound took numerous forms: one study used Chinese patent medicine of single-dose BAI, three studies used fixed prescriptions based on *S. baicalensis*, and three studies used a well-known traditional Chinese herbal prescription (Huangqin Decoction). The design of these clinical trials was as follows: six studies [26–31] compared the combined use of *S. baicalensis* and TACE with that of TACE alone: one study [32] compared the efficacy of *S. baicalensis* and that of basic therapy. All medicines in our research were fixed prescriptions and traditional Chinese patents with no individualizing drugs. Following the International Consolidated Standards of Reporting Trials reporting guidelines for CHM formulas [20], we extracted intervention details derived from the original literature, such as compound name and dosage form, representation of herb name in two languages, herbal sites, and dose; authentication method; formulating principle; pharmacology; prescription production; quality control; safety testing; and route of administration. This detailed list served as a checklist for the efficacy and safety assessments of CHM Formula RCTs (Supplementary Table S3).

#### 3.2.2. Preclinical study

Seventeen randomized controlled animal experimental studies and 31 *in vitro* studies were selected for inclusion. Although our entry criteria had unlimited genera and species, all preclinical studies in the final sample, except one, associated with Sprague Dawley rats [33], were mice of four types including Kunming species [34–40], ICR [41–43], C57BL/6 [44] and BALB/c-nude [15,40,45–47]. Females were used in four studies, males were used in nine, and combinations of males and females were used in the remaining four studies.

The body weight of the mice was 14–22 g. The body weight of SD rats was 180–210 g. Multiple forms of intervention of *S. baicalensis* existed against transplanted tumors in animal models, including *S. baicalensis*-extract [34], BAE [35,41,43,45,48], BAI [35,36,38–40], WOG [37,46,47], and *S. baicalensis*-based preparation [15,33,42,44]. The control group in some of the studies was the positive control of chemotherapy drugs, aiming to compare the treatment difference between chemotherapy drugs and *S. baicalensis*. Some researchers looked at the effects of different concentrations of *S. baicalensis*. There were four routes of administration; the most common was intragastric administration, followed by intraperitoneal injection. The characteristics of the 17 preclinical studies are presented in Supplementary Table S4.

### 3.3. Risk of bias

The bias in the seven selected studies was evaluated. Unfortunately, the methodological quality of most of the studies was low. The risk of bias is shown in Supplementary Figure S1. Four experiments described random methods for numerical random tables, one experiment generated random sequences using a statistical software, and two experiments were considered random. None of the experiments described the assigned blinding. Liang et al. described the blindness of the placebo in the experiment. No follow-up was mentioned in the experiments conducted by Liu and Wang. Wang’s trial, published in 2022, did not report the desired outcome measure for solid tumor efficacy. No other bias was observed in any of the studies based on the experience of the evaluators (Supplementary Figure S2).

### 3.4. Evidence of primary clinical outcome

The primary clinical outcome was the objective response rate (ORR), defined as the ratio of complete response (CR) plus partial response (PR) to the overall tumor response by RECIST version 1.1. We identified six RCTs [26–29,31,32] including 596 participants with the primary outcome measurement of ORR. The meta-analysis found insignificant heterogeneity in the results between individual studies (I^2^ = 0%) and fixed effect models (Risk ratio (RR) = 1.57, 95%CI: [1.30, 1.90], *p* < 0.00001) (Table 1). *Scutellaria. baicalensis* combined with TACE or basic treatment could lead to a significantly improved tumor response, and eventually, to superior clinical efficacy. Differences between the groups were significant.

**Table 1.** Meta-analysis of clinical outcomes.

Study or subgroup	Experimental		Control		Risk Ratio	
	Events	Total	Events total		M-H fixed 95% Cl	*p* value
CHM compound based S. baicalensis						
ORR						
Wang, et al.. 2016	18	30	9	30	2.00 [1.08, 3.72]	_
Li, et al.. 2019	38	56	27	56	1.41 [1.02, 1.95]	_
Liang, et al.. 2020	15	40	5	40	3.00 [1.20, 7.47]	_
Liu, et al.. 2013	6	32	4	32	1.50 [0.47, 4.82]	_
Wang, et al.. 2022	30	44	20	44	1.50 [1.02, 2.20]	_
Zhang, et al.. 2018	31	46	23	46	1.35 [0.95, 1.92]	_
Total (95% Cl) I^2^ = 0%					1.57 [1.30, 1.90]	*p* < 0.00001
KPS score						
Liu, et al.. 2013	30	32	20	32	1.50 [1.13, 1.99]	_
Zhang, et al.. 2018	35	46	30	46	1.17 [0.89, 1.52]	_
Total (95% Cl) I^2^ = 38%					1.32 [1.03, 1.69]	*p* = 0.03
Adverse events						
Wang, et al.. 2016	8	30	17	30	0.47 [0.24, 0.92]	_
Li, et al.. 2019	1	56	1	56	1.00 [0.06,15.59]	_
Wang, et al.. 2022	5	60	9	60	0.56 [0.20, 1.56]	_
Zhang, et al.. 2018	14	46	21	46	0.67 [0.39, 1.14]	_
Total (95% Cl) I^2^ = 0%					0.58 [0.40, 0.86]	*p* = 0.006

### 3.5. Evidence of secondary clinical outcome

#### 3.5.1. Assessment of life quality

The KPS score was used to evaluate quality of life, with a score increase of 10 points, and no change was calculated as valid. Two trials [26,28] reported KPS scores. The heterogeneity testing results included the statistical test value Q (*p* = 0.03) and I^2^ of 38% (moderate heterogeneity), with little overlap between the confidence intervals of a single study (Table 1). Heterogeneity was observed among the trials. The inclusion of studies was strictly in accordance with the criteria, and the possibility of clinical heterogeneity was small. Two articles with different risks of bias had different qualities, which may have led to methodological heterogeneity.

#### 3.5.2. Adverse events

Common adverse drug reactions in oncology medicine include marrow suppression, hepatic and renal dysfunction, and gastrointestinal disorders. Gastrointestinal symptoms were extracted from four studies. The analysis indicated that the risk rate of gastrointestinal symptoms of *S. baicalensis* combined with TACE or basic treatment was lower than that of the control group. The difference between each group was significant (*p* = 0.006) in a fixed-effects model (RR = 0.58, 95% CI: [0.40, 0.86], I^2^ = 0%) (Table 1).

### 3.6. *Evidence of primary preclinical outcome* in vivo

A meta-analysis including 12 preclinical studies [34–43,47,48] evaluated the efficacy of *S.baicalensis* in the treatment of HCC. The analysis indicated that any form of *S. baicalensis* component intervention significantly reduced the weight of transplanted tumors, regardless of BAE, WOG, or BAI S. baicalensis-based compound extract (BAE group: SMD = −4.80,95%CI: [–6.66, − 2.95], *p* < 0.00001; BAI group: SMD = –2.28,95%CI: [ −3.26, −1.30], *p* < 0.00001; *S. baicalensis* group: SMD = −0.86,95%CI: [–1.20, −0.53], *p* < 0.00001; WOG group: SMD = –1.41,95%CI [–2. 26, −0.57], *p* < 0.00001) (Table 2).

**Table 2.** Meta-analysis of preclinical outcome *in vivo* studies.

	Experimental			Control			Std. mean difference	
Study or subgroup	Mean	SD	Total	Mean	SD	Total	IV. Random. 95% Cl	P value
**Weight of tumor**								
**Baicalein**								
Cui, 2012-H	0.61	0.18	10	1.59	0.28	10	−3.99 [–5.62, −2.35]	_
Cui, 2012-M	0.88	0.24	10	1.59	0.28	10	−2.61 [–3.87, −1.35]	_
Cui, 2012-L	1.03	0.37	10	1.59	0.28	10	−1.63 [–2.68, −2.59]	_
Chiu, 2011-M	0.3	0.09	8	1.25	0.05	8	−12.34 [–17.36, −7.32]	_
Chiu, 2011-H	0.28	0.1	8	1.25	0.05	8	−11.60 [–16.33, −6.87]	_
Chiu, 2011-L	0.53	0.11	8	1.25	0.05	8	−7.97 [–11.29, −4.64]	_
Zheng, 2014-H	0.21	0.06	6	0.98	0.15	6	−6.22 [–9.46, −2.98]	_
Zheng, 2014-L	0.4	0.07	6	0.98	0.15	6	−4.57 [–7.08, −2.07]	_
Feng, 2012	0.77	0.88	10	1.01	0.15	10	−0.36 [–1.25, 0.52]	_
Subtotal (95% Cl) I^2^ = 89%							−4.80 [–6.66, −2.95]	*p* < 0.00001
**Baicalin**								
Cui, 2008-H	0.61	0.18	12	1.59	0.28	12	−4.02 [–5.50, −2.54]	_
Cui, 2008-M	0.88	0.24	12	1.59	0.28	12	−2.63 [–3.77, −1.49]	_
Cui, 2008-L	1.03	0.37	12	1.59	0.28	12	−1.65 [–2.60, −0.70]	_
Dong, 2013	0.5	0.03	10	0.73	0.04	10	−6.23 [–8.56, −3.90]	_
Lu, 2020-H	1.91	1.47	10	5.5	3.34	10	−1.33 [–2.32, −0.34]	_
Lu, 2020-M	2.08	1.54	10	5.5	3.34	10	−1.26 [–2.24, −0.28]	_
Lu, 2020-L	2.75	2.93	10	5.5	3.34	10	−0.84 [–1.76, 0.08]	_
Subtotal (95% Cl) I^2^ = 81%							−2.28 [–3.26, −1.30]	*p* < 0.00001
**Huangqin**								
Cheng, 2018	0.64	0.32	10	1.17	0.36	10	−1.49 [–2.51, −0.47]	_
Li-2, 2016-M	1.03	0.46	12	1.7	0.56	12	−1.26 [–2.15, −0.37]	_
Li-1, 2016-M	1.13	0.64	10	1.77	0.41	10	−1.14 [–2.10, −0.18]	_
Li-2, 2016-L	1.35	0.39	12	1.7	0.56	12	−0.70 [–1.53, 0.13]	_
Li-2, 2016-H	1.39	0.31	12	1.7	0.56	12	−0.66 [–1.49, 0.16]	_
Li-1, 2016-H	1.48	0.63	10	1.77	0.41	10	−0.52 [–1.42, 0.37]	_
Li-1, 2016-L	1.48	0.66	10	1.77	0.41	10	−0.51 [–1.40, 0.39]	_
Subtotal (95% Cl) I^2^ = 0%							−0.86 [–1.20, −0.53]	*p* < 0.00001
**Wogonin**								
Xu, 2013-H	0.52	0.18	6	1.64	0.61	6	−2.30 [–3.89, −0.71]	_
Xu, 2013-M	0.82	0.33	6	1.64	0.61	6	−1.54 [–2.90, −0.18]	_
Xu, 2013-L	1.25	0.25	6	1.64	0.61	6	−0.77 [–1.97, 0.42]	_
Subtotal (95% Cl) I^2^ = 82%							−1.41 [–2.26, −0.57]	*p* < 0.00001
Total (95% Cl) I^2^ = 87%							−2.14 [-2.70, −1.58]	*p* < 0.00001
**Apoptosis of tumor**								
**Baicalein**								
Cui, 2012-L	15.3	6.7	10	2.9	0.3	10	2.50 [1.27, 3.74]	_
Cui, 2012-M	24.6	3.1	10	2.9	0.3	10	2.61 [6.06, 12.82]	_
Cui, 2012-H	39.1	4.9	10	2.9	0.3	10	1.63 [6.42, 13.55]	_
Du, 2020-L	15.26	3.87	10	8.79	2.35	10	12.34 [0.83, 3.04]	_
Du, 2020-M	35.78	4.45	10	8.79	2.35	10	11.60 [4.60, 9.93]	_
Du, 2020-H	54.38	5.09	10	8.79	2.35	10	11.01 [7.11, 14.92]	_
Subtotal (95% Cl) I^2^ = 91%							6.63 [3.76, 9.50]	*p* < 0.00001
**Baicalin**								
Dong, 2013	13.6	1.1	10	2.2	1.9	10	7.03 [4.45, 9.62]	_
Subtotal (95% Cl)							7.03 [4.45, 9.62]	*p* < 0.00001
Total (95% Cl) I^2^ = 90%							6.68 [4.07, 9.29]	*p* < 0.00001
**Body weight**								
**Baicalein**								
Zheng, 2014-L	30.6	1.3	6	31.33	2.08	6	−0.73 [–2.69, 1.23]	_
Zheng, 2014-H	30.51	1.33	6	31.33	2.08	6	−0.82 [–2.80, 1.16]	_
Subtotal (95% Cl) I^2^ = 0%							−0.77 [–2.17, 0.62]	*p* = 0.28
**Baicalin**								
Lu, 2020-L	34.41	5.69	10	35.76	3.96	10	−1.35 [–5.65, 2.95]	_
Lu, 2020-M	34.45	5.1	10	35.76	3.96	10	−1.31 [–5.31, 2.69]	_
Lu, 2020-H	36.27	6.7	10	35.76	3.96	10	0.51 [–4.31,5.33]	_
Subtotal (95% Cl) I^2^ = 81%							−0.83 [–3.34, 1.67]	*p* = 0.51
**S. baicalensis**								
Li-1, 2016-L	31.2	3.9	10	31.9	2.4	10	−0.70 [–3.54, 2.14]	_
Li-1, 2016-M	30.1	2.9	10	31.9	2.4	10	−1.80 [–4.13, 0.53]	_
Li-1, 2016-H	30.7	2.5	10	31.9	2.4	10	−1.20 [–3.35, 0.95]	_
Li-2, 2016-L	34.1	4.2	12	33.9	2.5	12	0.20 [–2.57, 2.97]	_
Li-2, 2016-M	33.8	2.7	12	33.9	2.5	12	−0.10 [–2.18, 1.98]	_
Li-2, 2016-H	34.6	2.0	12	33.9	2.5	12	0.70 [–1.11, 2.51]	_
Yan, 2010	396.41	61.3	23	357.57	74.84	18	38.84 [–3.86, 81.54]	_
Yang, 2021	89.68	3.88	5	92.78	7.33	5	−3.10 [–10.37, 4.17]	
Subtotal (95% Cl) I^2^ = 6%							−0.41 [–1.32, 0.50]	*p* = 0.37
**Wogonin**								
Hong, 2020-1	21.32	1.4	6	23.1	1.14	6	−1.78 [–3.22, −0.34]	_
Hong, 2020-2	23.74	1.28	6	23.1	1.14	6	0.64 [–0.73, 2.01]	_
Xu, 2013-1	23.6	2.92	6	25.45	2.07	6	−1.85 [–4.71, 1.01]	_
Xu, 2013-2	24.09	2.21	6	25.45	2.07	6	−1.36 [–3.78, 1.06]	_
Xu, 2013-3	25.08	2.07	6	25.45	2.07	6	−0.37 [–2.71, 1.97]	_
Subtotal (95% Cl) I^2^ = 41%							−1.41 [–2.26, −0.57]	*p* = 0.10
total (95% Cl) I^2^ = 0%							−0.61 [–1.16, −0.07]	*p* = 0.03
**Liver index**								
**Baicalein**								
Zheng, 2014-L	7.41	0.39	6	7.56	0.19	6	−0.45 [–1.60, 0.70]	_
Zheng, 2014-H	7.16	0.41	6	7.56	0.19	6	−1.16 [–2.42, 0.11]	_
Subtotal (95% Cl) I^2^ = 0%							−0.77 [–1.62,0.08]	*p* = 0.08
**Baicalin**								
Dong, 2013	2.98	0.41	10	2.51	0.35	10	1.18 [0.21,2.15]	_
Subtotal (95% Cl)							1.18 [0.21,2.15]	*p* = 0.02
**S. baicalensis**								
Li-1, 2016-L	8.11	2.04	10	8.84	0.83	10	−0.45 [–1.34, 0.44]	_
Li-1, 2016-M	8.31	1.32	10	8.84	0.83	10	−0.46 [–1.35, 0.43]	_
Li-1, 2016-H	8.2	0.74	10	8.84	0.83	10	−0.78 [–1.70, 0.14]	_
Li-2, 2016-L	6.4	1.39	12	6.48	0.45	12	−0.07 [–0.88, 0.73]	_
Li-2, 2016-M	6.76	0.72	12	6.48	0.45	12	0.45 [–0.36, 1.26]	_
Li-2, 2016-H	6.42	0.36	12	6.48	0.45	12	−0.14 [–0.94, 0.66]	_
Yan, 2010	6.71	0.51	8	8.84	0.53	6	−3.85 [–5.83, −1.86]	_
Subtotal (95% Cl) I^2^ = 65%							−0.31 [–0.66, 0.03]	*p* = 0.07
total (95% Cl) I^2^ = 68%							−2.14 [–2.70, −1.58]	*p* = 0.14
**Spleen index**								
**Baicalein**								
Cui, 2012-L	5.45	0.37	10	5.12	0.28	10	0.96 [0.03, 1.90]	
Cui, 2012-M	8.04	0.34	10	5.12	0.28	10	8.98 [5.75,12.21]	
Cui, 2012-H	8.53	0.53	10	5.12	0.28	10	7.71 [4.90,10.51]	
Zheng, 2014-L	0.85	0.07	6	0.84	0.09	6	0.11 [–1.02, 1.25]	
Zheng, 2014-H	0.94	0.01	6	0.84	0.09	6	1.44 [0.11,2.77]	
Subtotal (95% Cl) I^2^ = 91%							3.34 [1.04, 5.64]	*p* = 0.004
**Baicalin**								
Lu, 2020-L	9.96	4.36	10	12.07	4.03	10	−0.48 [–1.37, 0.41]	
Lu, 2020-M	6.88	2.68	10	12.07	4.03	10	−1.45 [–2.46, −0.44]	
Lu, 2020-H	6.61	3.04	10	12.07	4.03	10	−1.46 [–2.48, −0.45]	
Subtotal (95% Cl) I^2^ = 29%							−1.09 [–1.76, −0.43]	*p* = 0.001
**Huangqin**								
Li-1, 2016-L	0.85	0.35	10	0.77	0.17	10	0.28 [–0.60, 1.16]	
Li-1, 2016-M	0.85	0.23	10	0.77	0.17	10	0.38 [–0.51, 1.27]	
Li-1, 2016-H	0.79	0.15	10	0.77	0.17	10	0.12 [–0.76, 1.00]	
Li-2, 2016-L	0.54	0.15	12	0.63	0.11	12	−0.66 [–1.49, 0.17]	
Li-2, 2016-M	0.54	0.1	12	0.63	0.11	12	−0.83 [–1.67, 0.01]	
Li-2, 2016-H	0.58	0.11	12	0.63	0.11	12	−0.44 [–1.25, 0.37]	
Yan, 2010	0.38	0.09	12	0.48	0.1	12	−1.01 [–1.87, −0.16]	
Subtotal (95% Cl) I^2^ = 39%							−0.32 [–0.73, 0.09]	*p* = 0.12
total (95% Cl) I^2^ = 85%							0.30 [–0.39,1.00]	*p* = 0.39
**Caspase-3**								
**Baicalein**								
Cui, 2012-L	142.54	13.36	10	100.67	4.45	10	4.03 [2.38, 5.67]	_
Cui, 2012-M	197.77	16.93	10	100.67	4.45	10	7.51 [4.77, 10.26]	_
Cui, 2012-H	280.62	28.51	10	100.67	4.45	10	8.45 [5.40, 11.50]	
Subtotal (95% Cl) I^2^ = 77%							6.45 [3.50, 9.40]	*p* < 0.0001
**Baicalin**								
Dong, 2013	31.31	6.09	10	20.08	5.01	10	1.93 [0.83, 3.03]	_
Subtotal (95% Cl)							1.93 [0.83, 3.03]	*p* = 0.00006
**S. baicalensis**								
Lam, 2015	8.32	3.39	5	0.68	0.54	5	2.84 [0.82, 4.87]	_
Yang, 2021	6.28	3.64	5	1.26	0.53	5	1.74 [0.16, 3.32]	_
Subtotal (95% Cl) I^2^ = 0%							−0.31 [–0.66, 0.03]	*p* = 0.00007
Total (95% Cl) I^2^ = 83%							−2.14 [–2.70, −1.58]	*p* < 0.0001
**VEGF**								
**Baicalein**								
Du, 2020-L	38.79	4.13	10	53.85	5.15	10	−3.09 [–4.47, −1.71]	
Du, 2020-M	23.84	3.37	10	53.85	5.15	10	−6.60 [–9.05, −4.16]	
Du, 2020-H	8.55	4.13	10	53.85	5.15	10	−9.29 [–12.63, −5.96]	
Subtotal (95% Cl) I^2^ = 86%							−6.09 [–9.71, −2.47]	*p* = 0.0010
**Huangqin**								
Li-2, 2016-L	91	4	12	100	0.01	12	−3.07 [–4.31, −1.83]	
Li-2, 2016-M	89	14	12	100	0.01	12	−1.07 [–1.94, −0.21]	
Li-2, 2016-H	47	9	12	100	0.01	12	−8.04 [–10.65, −5.43]	
Subtotal (95% Cl) I^2^ = 93%							−3.79 [–6.75, −0.82]	*p* = 0.01
Total (95% Cl) I^2^ = 91%							−4.85 [–7.04, −2.66]	*p* < 0.0001

### 3.7. *Evidence of secondary preclinical outcome* in vivo

#### 3.7.1. Apoptosis of tumor cells

Only two studies involved the use of BAE. The mention of apoptosis of tumor cells after hematoxylin and eosin staining was reported in another study. The results of these three studies [35,40,45] indicated that *S. baicalensis* increased the apoptosis of tumor cells (SMD = 6.68, 95%CI: [4.07, 9.29], *p* < 0.00001) (Table 2). In summary, *S. baicalensis* and its active components inhibited liver cancer in animals.

#### 3.7.2. Body weight after treatment

Our analysis was based on eight studies [33,38,39,42,43,46,47] related to outcomes in relation to body weight after treatment. There was no significant difference in body weight after treatment between the intervention and control groups (BAE group: SMD = –0.77, 95%CI: [–2.17, 0.62], *p* = 0.28; BAI group: SMD = –0.83, 95% CI: [–3.34, 1.67], *p* = 0.51; *S. baicalensis* group: SMD = –0.41, 95%CI: [- 1.32, −0.50], *p* = 0.37; WOG group: SMD = –0.70, 95%CI: [–1.52, 0.12], *p* = 0.10). Compared to the model group, there was a difference in body weight after treatment (SMD = –2.41, 95%CI: [–2.70, −1.58], *p* = 0.03) (Table 2). All the results were significant.

#### 3.7.3. Liver, kidney, and spleen index

We identified five studies [33,40,42,43] reporting the liver index (BAE group: SMD = –0.77, 95% CI: [–1.62, 0.08], *p* = 0.08; BAI group: SMD= 1.18, 95% CI [0.21, 2. 15], *p* = 0.02; *S. baicalensis* group: SMD = –0.31, 95% CI: [–0.66, 0.03], *p* = 0.07; Total: SMD = –0.23, 95% CI: [–0.53, 0.07], *p* = 0. 14) and six studies [33,35,38,39,42,43] reporting the spleen index (BAE group: SMD = 3.34, 95% CI [1.04, 5.64], *p* = 0.004; BAI group: SMD = –1.09, 95% CI: [- 1.76, −0.43], *p* = 0.001; *S. baicalensis* group: SMD = –0.32, 95% CI: [–0.73, 0.09], *p* = 0.12; Total: SMD = 0.30, 95% CI: [–0.39, 1.00], *p* = 0.39) that were recorded and calculated at the time of animal sacrifice for *S. baicalensis* interventions versus normal saline only. No pooled kidney index could be analyzed with only one experiment [40] reporting kidney weight at the time of sacrifice. As shown in Table 2, there were no significant differences in the liver and spleen indices between the *S. baicalensis* and control groups.

#### 3.7.4. Caspase-3

Table 2 shows the combined results based on four studies [35,40,44,49] of apoptosis-related protein expression levels of caspase-3 in *S. baicalensis* versus the control group (SMD = 4.09, 95% CI: [2.25, 5.93], *p* < 0.0001). Pooled analysis showed that *S. baicalensis* had significantly better Caspase-3 apoptosis than the control in preclinical animal studies.

#### 3.7.5. VEGF

Vascular endothelial growth factor in the tumor tissue of xenografts of human liver cancer cell lines was determined by immunoassay in Du. et al.’s research [45]. Thus, the meta-analysis showed that *S. baicalensis*-based compounds were more effective than the group of models in improving the expression of VEGF in mice (SMD = –30.12, 95% CI: [–46.84 to −13.41], *p* = 0.0004) (Table 2).

### 3.8. *Evidence of preclinical outcome* in vitro

#### 3.8.1. Cell proliferation

In cell cycle experiments, the results of three studies [43,46,50] showed that *S. baicalensis* induced cell cycle arrest. Compared to the control group, BAE and WOG were associated with increased expression of the cell cycle-associated [36] protein, CyclinD1 (SMD = −0.27, 95% CI: [–0.46, − 0.07], *p* = 0.007). As expected, liver cancer cell inhibition was observed (Table 3).

**Table 3.** Meta-analysis for Bcl-2, MMP-2, MMP-9, caspase-3, and CyclinD1 (*in vitro* studies).

	Experimental			Control			Std. Mean Difference	
Study or Subgroup	Mean	SD	Total	Mean	SD	Total	IV. Random. 95% Cl	P value
**Bcl-2**								
**Baicalein**								
Cui, 2008	32.21	3.35	3	59.26	4.45	3	−5.70 [–11.43, 0.03]	_
Subtotal (95% Cl)							−5.70 [–11.43, 0.03]	*p* = 0.05
**Wogonin**								
Liu, 2016-H	0.32	0.02	3	0.99	0.03	3	−21.02 [–41.39, −0.66]	_
Liu, 2016-L	0.88	0.02	3	0.99	0.03	3	−3.45 [–7.15, 0.25]	_
Liu, 2016-M	0.67	0.01	3	0.99	0.03	3	−11.45 [–22.62, −0.28]	_
Subtotal (95% Cl) I^2^ = 54%							−4.73 [–8.18, −1.27]	*p* = 0.007
Total (95% Cl) I^2^ = 32%							−4.99 [–7.95, −2.02]	*p* = 0.001
**MMP-2**								
**Baicalein**								
Chiu, 2011	0.58	0.25	3	0.99	0.12	3	−1.67 [–3.95, 0.60]	_
Subtotal (95% Cl)							−1.67 [–3.95, 0.60]	*p* = 0.15
**S. baicalensis**								
Park, 2014-H	0.13	0.11	3	0.79	0.01	3	−6.76 [–13.48, −0.04]	_
Park, 2014-L	0.42	0.13	3	0.79	0.01	3	−3.21 [–6.70, 0.28]	_
Subtotal (95% Cl) I^2^ = 0%							−3.96 [–7.06, −0.87]	*p* = 0.01
**Wogonin**								
Liu, 2016-H	0.08	0.01	3	0.99	0.01	3	−72.80 [–143.11, −2.49]	_
Liu, 2016-L	0.38	0.01	3	0.99	0.01	3	−48.80 [–95.95, −1.65]	_
Liu, 2016-M	0.34	0.02	3	0.99	0.01	3	−32.89 [–64.68, −1.09]	_
Subtotal (95% Cl) I^2^ = 0%							−42.17 [–66.85, −17.48]	*p* = 0.0008
Total (95% Cl) I^2^ = 62%							−2.69 [–4.52, −0.87]	*p* = 0.004
**MMP-9**								
**Baicalein**								
Chiu, 2011	0.37	0.13	3	0.99	0.12	3	−3.96 [–8.11, 0.18]	_
Subtotal (95% Cl)							−3.96 [–8.11, 0.18]	*p* = 0.06
**Baicalin**								
Xiao, 2012-H	21.85	3.41	3	41.48	3.55	3	−4.51 [–9.15, 0.13]	_
Xiao, 2012-L	33.26	4.07	3	41.48	3.55	3	−1.72 [–4.03, 0.59]	_
Subtotal (95% Cl) I^2^ = 10%							−2.27 [–4.34, −0.21]	*p* = 0.03
Total (95% Cl) I^2^ = 0%							−2.61 [–4.46, −0.76]	*p* = 0.006
**Caspase-3**								
**Baicalein**								
Kuo, 2009	0.79	0.05	3	0.19	0.03	3	0.60 [0.53, 0.67]	_
Liang, 2012-H	3.55	0.19	3	0.99	0.01	3	2.56 [2.34, 2.78]	_
Liang, 2012-L	1.5	0.42	3	0.99	0.01	3	0.51 [0.03, 0.99]	_
Liang, 2012-M	2.91	0.52	3	0.99	0.01	3	1.92 [1.33, 2.51]	_
Total (95% Cl) I^2^ = 99%							1.40 [0.18, 2.61]	*p* = 0.02
**CyclinD1**								
**Baicalein**								
Zheng, 2014-H	0.33	0.07	3	1	0.01	3	−0.67 [–0.75, −0.59]	
Zheng, 2014-L	0.21	0.1	3	1	0.01	3	−0.79 [–0.90, −0.68]	
Subtotal (95% Cl) I^2^ = 65%							−0.72 [–0.84, −0.61]	*p* < 0.00001
**Wogonin**								
Hong, 2020-H	3.99	0.64	3	1.01	0.13	3	2.98 [2.24, 3.72]	_
Hong, 2020-L	2.3	0.4	3	1.01	0.13	3	1.29 [0.81, 1.77]	_
Liu, 2016-H	0.28	0.01	3	1	0.01	3	−0.72 [–0.74, −0.70]	_
Liu, 2016-L	0.47	0.02	3	1	0.01	3	−0.53 [–0.56, −0.50]	_
Liu, 2016-M	0.67	0.01	3	1	0.01	3	−0.33 [–0.35, −0.31]	_
Subtotal (95% Cl) I^2^ = 100%							−0.02 [–0.27, 0.22]	*p* = 0.84
Total (95% Cl) I^2^ = 100%							−0.27 [–0.46, −0.07]	*p* = 0.007

#### 3.8.2. Cell apoptosis

In the western blot analysis of apoptosis-related factors, the meta-analysis results of two cell experiments [36,50] showed that the suppressive effects of BAE and WOG on Bcl-2 expression were significant (SMD = −4.99 95% CI: [-7.95, −2.02], *p* = 0.001). Another two [51,52] were included in the meta-analysis to confirm that BAE promoted pro-apoptotic caspase-3 enzyme activity (SMD = 1.40, 95% CI: [0.18, 2.61], *p* = 0.02) (Table 3).

#### 3.8.3. Cell invasion and metastasis

In ELISA and western blotting experiments of protein activity assays, the outcomes of three meta-analyses [48,50,53] showed significantly decreased expression of matrix metalloproteinase 2 (MMP-2) in *S. baicalensis* compared to that in the control group (SMD = −2.69, 95% CI: [-4.52, −0.87], *p* = 0.004). Additional meta-analyses of two studies [48,54] indicated that BAE and BAI significantly decreased MMP-9 expression (SMD = −2.61, 95% CI: [-4.46, −0.76], *p* = 0.006). In the tumor extracellular matrix (ECM), MMP is suppressed, which prejudices the invasion and metastasis of tumor cells (Table 3).

## 4. Discussion

Surgical resection and liver transplantation are the mainstay of radical treatments for HCC [1]. The prime time for patient recovery has been missed because of high heterogeneity, rapid progression, and non-obvious early symptoms. The curative options for patients with stage II, III or IV is TACE, ablation, and systemic therapy, although dramatically prolonging survival by several months, the prognosis is still dismal [55–57]. In China, CHM is a traditional treasure because of the popular use of unique and novel complementary strategies for liver cancer.

*Scutellaria. baicalensis*, a herbal medicine with over 1800 years of history, is used for digestive tract cancers and possesses multi-fold anti-tumor and immunoregulatory properties [6]. Professor Yung-Chi Cheng’s team explored Huangqin decoction (PHY906)-assisted chemotherapy-induced toxicities [58]. Liu et al. designed an open-label clinical trial collecting patients from five medical centers, and assessed that a combination of 600, 800, and 1000 mg/bid PHY906 and capecitabine for the treatment of diarrhea, nausea, and vomiting (related to chemotherapeutic toxicities) was safe and effective [59]. Yun et al. verified that PHY906 differed for each race, with a more beneficial effect on the median survival time for Asians [60]. Zhang et al. found Huangqin-decoction could cure diarrhea with sorafenib by adjusting the function of intestinal flora [61]. Increasing evidence has highlighted the advantages of CHM in relieving adverse reactions (related to TACE, chemotherapy, and immunotherapy) and improving cancer cachexia, consistent with our results. The anti-cancer effect of Scutellaria. baicalensis was found in clinical experiences with the condition of taking humans as a research object. However preclinical research related to the exploration of drug function and action mechanism. In the era of evidence-based medicine and with the surging number of publications, a meta-analysis of preclinical could reduce bias and random error between studies, avoid the reuse of laboratory animals and cells, and decrease the risk of translating experimental results into the clinic.

To the best of our knowledge, this is the first systematic review and meta-analysis to demonstrate that *S. baicalensis* and its bioactive compounds have therapeutic potential and mechanisms of action against HCC from multiple perspectives, including efficacy, safety, and plausible molecular mechanisms. Seven clinical RCTs involving 646 individuals revealed that CHM preparation with *S. baicalensis* as the sovereign herb exerted therapeutic effects on the enhancement of tumor response, improvement of quality of life-based on KPS, and decrease of chemotherapy drug-related toxicity. Based on the existing literature we selected, in the clinical setting, combined therapy was more often conducted as a union of *S. baicalensis*-based compounds and TACE, compared with other combinations. In our results, we identified 17 preclinical studies *in vivo* and thirty-one *in vitro* studies, including 553 animal models and ten cell types. In order to augment the strength of our evidence, we used fixed-effect and random-effect models to evaluate the degree and mechanism of actions that are accountable for the anticancer effects of the bioactive compounds of *S. baicalensis*, including BAE, BAI, and WOG. In terms of results, there was no significant difference between the two models (Supplementary Table S5-S6). As reported, *S. baicalensis* acts as a tumor suppressor in multiple *in vitro* and *in vivo* HCC models. The investigation of the binding mechanism of *S. baicalensis* with molecular targets is currently underway, as the possible action pathways were apoptosis of tumor-promoting cells and inhibition of cell proliferation, as well as autophagy induction and inhibition of tumor angiogenesis and lymphangiogenesis.

Cell cycle regulation is an important tumor suppressor mechanism that inhibits cell proliferation. Wogonin and BAE induce HCC cell cycle arrest by blocking the G0/G1 phase, or reducing the distribution of the S and G2/M phases, and impairing cancer cell proliferation [43,46,51]. Smooth progression of the cell cycle requires the interaction of multiple factors such as cyclin, cyclin-dependent kinase (CDK), and cyclin-dependent kinase inhibitors. Baicalin and BAE activate cell cycle regulation through inhibition of the expression and function of cell cycle genes, including CyclinE, CyclinD1, CyclinB1, CyclinA2, and CDK, by blocking the PI3K/AKT/mTOR and AMPK signaling pathways [40,62–64].

Apoptosis is a potent way of programmed cell death, which is also applied to tumor cells. *Scutellaria. baicalensis* plays a role in apoptosis-related signaling molecules, such as Bcl-2 family proteins, cytochrome C, and caspases. Bax is an apoptosis-promoting gene and Bcl-2 is an apoptosis-suppressor gene. The expression of Bax requires activation of the Ras/Raf/MEK/ERK pathway, involving the accession of the tumor suppressor gene, p53. Baicalein affects apoptosis by inhibiting the expression of Bcl-2/Bax [65–67]. Caspases were downstream effectors of the Bcl-2/Bax pathway. Baicalein and WOG can induce caspases regulated by the UPR and PI3K/ERK/AKT pathways [36,47,50,66]. Supporting evidence of caspase activation related to mitochondrial membrane permeability has been reported in the literature [47,68]. Several studies have investigated the effects of autophagy on apoptosis. Baicalein and BAI inhibit the expression of LC-3I, LC-3II, Atg5, CD147, and Beclin 1, which regulate important events during the activation of autophagy [69,70]. Another apoptotic mechanism is an activation of endoplasmic reticulum (ER) stress, there is evidence that BAI activates the ATF6 signaling pathway, thereby catalyzing ER stress [71].

With the sustained release of intercellular gap junctions and the expression of Cell surface adhesion molecules, tumor cells acquire invasion and metastasis. This process could be hindered by the action of BAI through upregulation of Cx26 and Cx43 expression and by the action of BAE through inhibition of Ezrin [41,72]. Degradation of extracellular matrix components requires matrix metalloproteinases (MMPs), a set of enzymes regulated by the transcription factors NF-KB, p38, MAPK, endogenous HMGB1, and the ROS pathway. Our study showed that BAE, BAI, and WOG could inhibit the activities of MMP-2 and MMP-9, which reasonably explains the inhibitory effect of *S. baicalensis* on tumor invasion and metastasis [48,53,54]. In the present study, based on the above anticancer mechanism of action *in vitro* and *in vivo*, we summarized the potential mechanism of action of BAI, BAE, and WOG in HCC treatment (Supplementary Table S7), as presented in Figure 1. The preclinical meta-analysis integrating the existing literature focuses on the the direct cytotoxic effects of *Scutellaria baicalensis* on liver cancer cells. The inhibition of proliferation, invasion, metastasis, and angiogenesis of *S.baicalensis* may be associated with multiple biological pathways, including the cell cycle, PI3K-Akt, Hippo, and Nf-KB pathways, as well as ROS, ER stress, autophagy, caspase-related apoptosis, and adhesion molecule release. In addition, the adjunctive anticancer effects of *S. baicalensis* are warrant additional research into regulating the tumor microenvironment to stimulate immune responses and binding PD-1/PD-L1 immune checkpoints to increase effects or decrease drug resistance.

More studies in the future, such as real-world observational studies, are needed to corroborate *S. baicalensis* as a candidate for HCC treatment and to explore the synergistic therapeutic effects of cancers and RCTs with low risk for bias, multi-center design, and large sample sizes to confirm the long-term prognosis of anticancer therapy.

## 5. Limitations

This study had some limitations. There was no unified standard for the composition, dosage, and quality of compound preparations, and it was difficult to evaluate their efficacy and safety. The problematic commonness of the selected studies was the small sample size and poor overall methodological quality, which reduced the reliability of the conclusions. Fortunately, a high-quality multicenter randomized placebo-controlled trial with 125 participants assessing high quality is in progress and is not yet available, the aim of which is to compare the efficacy and safety of YIV-906 plus sorafenib versus sorafenib alone as a first-line systemic treatment for patients with hepatitis B (+)-associated advanced HCC [73]. In the future, we plan to update our systematic review and meta-analysis by including the data from this trial. In addition, none of the included studies mentioned the implementation and acceptance of allocation concealment by both participants and statisticians, which may have a high risk of bias. Moreover, the articles had limited screening. Finally, unpublished data were excluded from this study.

## 6. Conclusions

In conclusion, the present study revealed that a CHM preparation with S. baicalensis as a sovereign herb combined with TACE or basic treatment exerts a safe and effective function in the treatment of HCC. Meanwhile, preclinical studies have shown that *S. baicalensis*, BAE, BAI, and WOG have potential therapeutic mechanisms, which include inhibiting cell cycle progression and proliferation, anti-angiogenesis, promoting apoptosis, and regulating the tumor microenvironment.

## Supplementary Material

Supplemental Material

## Data Availability

All authors confirm that the data supporting the findings of this study are available within the article and/or its supplementary materials
