# Supplementary material for: Evidence and possible mechanism of Scutellaria baicalensis and its bioactive compounds for hepatocellular carcinoma treatment
Source: Ann Med. 2024 Jan 17;55(2):2247004. doi: 10.1080/07853890.2023.2247004 (PMC10795786; doi:10.1080/07853890.2023.2247004)
Supplement: Supplemental Material [file IANN_A_2247004_SM6826.zip › Table_S1.docx]

| Date base | Search Math | [Article](javascript:;) |
| --- | --- | --- |
| CNKI | （主题：肝癌（精确）) OR （篇关摘：肝细胞癌 ＋ 原发性肝癌 ＋ 肝肿瘤 + 肝内胆管癌(精确)) AND ((主题：黄芩（精确）) OR （篇关摘：黄芩 ＋ 黄芩素 ＋ 黄芩苷 ＋ 汉黄芩素（模糊）)) | 406 |
| VIP | 肝癌 or 原发性肝癌 or 肝肿瘤 or 肝细胞癌  黄芩 or 黄芩素 or 汉黄芩素 or 黄芩苷 | 4 |
| Wanfang | (主题:(肝癌) or 题名或关键词:(肝肿瘤 or 原发性肝癌 or 肝细胞癌 or 肝内胆管癌)) and (主题:(黄芩) or 题名或关键词:(黄芩素 or 汉黄芩素 or 黄芩苷)) | 343 |
| sinomed | ((("肝癌"[常用字段] OR "肝肿瘤"[常用字段] OR "肝脏肿瘤"[常用字段] OR "肝部肿瘤"[常用字段] OR "肝肿瘤"[主题词]) OR ("肝肿瘤"[常用字段] OR "肝脏肿瘤"[常用字段] OR "肝部肿瘤"[常用字段] OR "肝癌"[常用字段] OR "肝肿瘤"[主题词]) OR ("肝细胞癌"[常用字段] OR "肝细胞瘤"[常用字段] OR "癌, 肝细胞"[主题词]) OR "原发性肝癌"[常用字段] OR ("肝内胆管癌"[常用字段] OR "胆管上皮癌"[常用字段] OR "胆管细胞癌"[常用字段] OR "肝外胆管癌"[常用字段] OR "胆管上皮癌"[主题词])) AND (("黄芩"[全部字段] OR "黄芩"[主题词]) OR "黄芩苷"[常用字段] OR ("汉黄芩素"[常用字段] OR "汉黄芩素"[主题词]) OR "黄芩素"[常用字段])) | 155 |
| Pubmed | ((((((((((((((((((("Carcinoma, Hepatocellular"[Mesh]) OR (Carcinomas, Hepatocellular[Title/Abstract])) OR (Hepatocellular Carcinomas[Title/Abstract])) OR (Liver Cell Carcinoma, Adult[Title/Abstract])) OR (Liver Cancer, Adult[Title/Abstract])) OR (Adult Liver Cancer[Title/Abstract])) OR (Adult Liver Cancers[Title/Abstract])) OR (Cancer, Adult Liver[Title/Abstract])) OR (Cancers, Adult Liver[Title/Abstract])) OR (Liver Cancers, Adult[Title/Abstract])) OR (Liver Cell Carcinoma[Title/Abstract])) OR (Carcinoma, Liver Cell[Title/Abstract])) OR (Carcinomas, Liver Cell[Title/Abstract])) OR (Cell Carcinoma, Liver[Title/Abstract])) OR (Cell Carcinomas, Liver[Title/Abstract])) OR (Liver Cell Carcinomas[Title/Abstract])) OR (Hepatocellular Carcinoma[Title/Abstract])) OR (Hepatoma[Title/Abstract])) OR (Hepatomas[Title/Abstract])) AND (((((((((((("Scutellaria baicalensis"[Mesh]) OR (Huang Qin[Title/Abstract])) OR (Skullcap, Baikal[Title/Abstract])) OR (Baikal Skullcap[Title/Abstract])) OR (Scutellariae radix[Title/Abstract])) OR (Huangqin[Title/Abstract])) OR (baicalin[Title/Abstract])) OR (7-D-glucuronic acid-5,6-dihydroxy-flavone[Title/Abstract])) OR (7-D-glucuronic acid-5,6-dihydroxyflavone[Title/Abstract])) OR (Baicalein[Title/Abstract])) OR (wogonin[Title/Abstract])) OR (5,7-dihydroxy-8-methoxyflavone[Title/Abstract])) | 97 |
| Cochrane Library | (MeSH descriptor: [Carcinoma, Hepatocellular] explode all trees or  (Carcinomas, Hepatocellular or Hepatocellular Carcinomas or Liver Cell Carcinoma, Adult or Liver Cancer, Adult or Adult Liver Cancer or Adult Liver Cancers or Cancer, Adult Liver or Cancers, Adult Liver or Liver Cancers, Adult or Liver Cell Carcinoma or Carcinoma, Liver Cell or Carcinomas, Liver Cell or Cell Carcinoma, Liver or Cell Carcinomas, Liver or Liver Cell Carcinomas or Hepatocellular Carcinoma)):ti,ab,kw and (MeSH descriptor: [Scutellaria baicalensis] explode all trees or (Scutellaria baicalensis or Huang Qin or Skullcap, Baikal or Baikal Skullcap or Scutellariae radix or Huangqin or baicalin or Baicalein or wogonin):ti,ab,kw | 1 |
| Web of science | (TS=(Carcinoma, Hepatocellular) OR AB=(Carcinomas, Hepatocellular OR Hepatocellular Carcinomas OR Liver Cell Carcinoma, Adult OR Liver Cancer, Adult OR Adult Liver Cancer OR Adult Liver Cancers OR Cancer, Adult Liver OR Cancers, Adult Live OR Liver Cancers, Adult OR Liver Cell Carcinoma OR Carcinoma, Liver Cell OR Carcinomas, Liver Cell OR Cell Carcinoma, Liver OR Cell Carcinomas, Liver OR Liver Cell Carcinomas OR Hepatocellular Carcinoma OR Hepatoma OR Hepatomas)) AND (TS=(Scutellaria baicalensis) OR AB=(Scutellaria baicalensis OR Huang Qin OR Skullcap, Baikal OR Baikal Skullcap OR Scutellariae radix OR Huangqin OR baicalin OR 7-D-glucuronic acid-5,6-dihydroxy-flavone OR 7-D-glucuronic acid-5,6-dihydroxyflavone OR Baicalein OR wogonin OR 5,7-dihydroxy-8-methoxyflavone)) | 162 |
| Embase | 'carcinomas, hepatocellular':ab,ti OR 'hepatocellular carcinomas':ab,ti OR 'liver cell carcinoma, adult':ab,ti OR 'liver cancer, adult':ab,ti OR 'adult liver cancer':ab,ti OR 'adult liver cancers':ab,ti OR 'cancer, adult liver':ab,ti OR 'cancers, adult live':ab,ti OR 'liver cancers, adult':ab,ti OR 'liver cell carcinoma':ab,ti OR 'carcinoma, liver cell':ab,ti OR 'carcinomas, liver cell':ab,ti OR 'cell carcinoma, liver':ab,ti OR 'cell carcinomas, liver':ab,ti OR 'liver cell carcinomas':ab,ti OR 'hepatocellular carcinoma':ab,ti OR hepatoma:ab,ti OR hepatomas:ab,ti and 'scutellaria baicalensis':ab,ti OR 'huang qin':ab,ti OR 'skullcap, baikal':ab,ti OR 'baikal skullcap':ab,ti OR 'scutellariae radix':ab,ti OR huangqin:ab,ti OR baicalin:ab,ti OR '7-d-glucuronic acid-5,6-dihydroxy-flavone':ab,ti OR '7-d-glucuronic acid-5,6-dihydroxyflavone':ab,ti OR baicalein:ab,ti OR wogonin:ab,ti OR '5,7 dihydroxy 8 methoxyflavone':ab,ti | 126 |
