# Supplementary material for: Evidence and possible mechanism of Scutellaria baicalensis and its bioactive compounds for hepatocellular carcinoma treatment
Source: Ann Med. 2024 Jan 17;55(2):2247004. doi: 10.1080/07853890.2023.2247004 (PMC10795786; doi:10.1080/07853890.2023.2247004)
Supplement: Supplemental Material [file IANN_A_2247004_SM6826.zip › Table_S2.docx]

**Supplementary Table 1** | The main characteristics of the clinical study

| Study ID | Information of sample | | | | | | Control | Intervention | | | Time | Outcome |
| --- | --- | --- | --- | --- | --- | --- | --- | --- | --- | --- | --- | --- |
|  | T/C | Female/Male | | Age | | HCC  staging | TACE/ basic  treatment | drug | dose | pathway |  |  |
| Liu,et al,2013 | 32/32 | 5/27 | 6/26 | 52.2±9.74 | 54.7±10.30 | phaseII/  III | L-OHP+MM C+HCPT+5-  FU | WD-2 | 200ml | oral | 8W | ①② |
| Wang,et al,2016 | 30/30 | 12/18 | 10/20 | 46.2±4.3 | 47.5±5.2 | N/A | DDP+RPI | Huangqingan  capsule | 0.5g | oral | 90d | ①③ |
| Zhang,et  al,2018 | 46/46 | 15/31 | 16/30 | 54.18±12.62 | 52.5±9.6 | N/A | MMC+ADM +5-FU | Yiqi  Yangyin  Decoction | 200ml | oral | 8w | ①②③ ④ |
| Li,et  al,2019 | 56/56 | 15/41 | 13/43 | 46.15±6.27 | 47.24±4.68 | N/A | DDP+EPI | Huangqin  Decoction | 200ml | oral | 8w | ①③ |
| Liang,et al.2020 | 40/40 | 14/26 | 19/21 | 51±8.53 | 52±7.12 | N/A | WeiRuiDe | Wenshen Decoction | 160ml | oral | 8w | ① |
| Wang,et  al.2022 | 44/44 | 14/30 | 13/31 | 58.8±7.8 | 59.5±4.6 | N/A | DDP+EPI | Huangqin Decoction | 200ml | oral | 8w | ① |
| Wang  and  Zhu,2022 | 60/60 | 22/38 | 21/39 | 46.54±5.23 | 46.74±5.33 | N/A | DDP+EPI | Huangqin Decoction | 200ml | Oral | 8w | ③ |

**Abbreviations：**T, treatment; C, control; L-OHP, oxaliplatin; DDP, cisplatin; MMC, mitomycin C; EPI, epirubicin; ADM, doxorubicin; 5-FU, 5-fluorouracil; Outcomes: ①ORR ②KPS ③gastrointestine ④myelosuppression.
