# Supplementary material for: Evidence and possible mechanism of Scutellaria baicalensis and its bioactive compounds for hepatocellular carcinoma treatment
Source: Ann Med. 2024 Jan 17;55(2):2247004. doi: 10.1080/07853890.2023.2247004 (PMC10795786; doi:10.1080/07853890.2023.2247004)
Supplement: Supplemental Material [file IANN_A_2247004_SM6826.zip › Table_S3.docx]

| **Table S1. Checklist of Items for Reporting Trials of Chinese Herbal Medicine Formulas** | | | | | |  |  | |  | |  | |  | |  | |  | |  |  |  |
| --- | --- | --- | --- | --- | --- | --- | --- | --- | --- | --- | --- | --- | --- | --- | --- | --- | --- | --- | --- | --- | --- |
| **Category** | **Study** | **Chinese medicine compound** | **Composition and dosage of Chinese medicine prescriptions (Chinese Pinyin name and Latin name)** | **Authentication method** | **Principles, rationale, and interpretation of forming the formula** | **Pharmacologic study results of the formula** | | **Production method of the formula** | | **Quality control of each ingredient and of the**  **product of the formula** | | **Safety assessment of the formula** | | **Dosage of the formula** | | **Administration Route** | | **Preparation method** | | | |
| Clinical research | Liu 2013 | WD-2 Decotion | Chaihu  Hungqin  Baizhu  Yujin  Zhiqiao  Fushen  Fuling  Chishao  Baishao  Chenpi  Danggui | N/A | Chaihu:Target angiogenin and VEGF.  Huangqin: Pro-apoptotic,anti-invasion and metastasi.  Baizhu：Liver-protection, immune regulatory, anti-inflammatory.  Zhiqiao, Chenpi, Yujin: anti-inflammatory.  Fushen, Fuling, Chishao, Danggui：enhances immune function. | N/A | | N/A | | N/A | | N/A | | bid, 8 w | | Oral | | Decoction | | | |
|  | Wang, et al. 2016 | Baicalin Capsules | Baicalin | N/A | Huangqin:induces cell cycle arrest and apoptosis. | N/A | | N/A | | N/A | | N/A | | tid, 90 d | | Oral | | Capsule | | | |
|  | Zhang, et al 2013 | Yiqi Yangyin  Decoction | Chaihu9g  Huangqin12g  Fabanxia10g  Dangshen12g  Danggui12g  Danshen12g  Gancao6g | N/A | Chaihu:Target angiogenin and VEGF.  Huangqin：Pro-apoptotic, anti-invasion, metastas and pro-necrosis.  Fabanxia：anti-inflammatory, DNA damage.  Dangshen, Danggui：Pro-apoptotic, anti-invasion and metastas, affect metabolism.  Danshen:Target angiogenin and VEGF, anti-inflammatory  Gancao:Immune regulatory | N/A | | N/A | | N/A | | N/A | | bid, 200 ml, 8w | | Oral | | Decoction | | | |
|  | Li, et al 2019 | Huangqin  Decoction | Huangqin 18 g  Baishao18 g  Gancao 10 g  Dazao 6g | N/A | Huangqin：Pro-apoptotic, anti-invasion and metastas, pro-necrosis.  Baishao：Pro-apoptotic, Immune regulatory, anti-inflammatory.Gancao:antibacterial, anti-inflammatory, hypoglycemic, lipid-lowering, antioxidant,  Dazao: antibacterial | N/A | | N/A | | N/A | | N/A | | bid, 200 ml, 8 w | | Oral | | Add 2,000 mL of water to decoct the traditional Chinese medicine compound to 400 mL of the remaining decoction. | | | |
|  | Liang, et al 2020 | Wenshen Decoction | Fuzi6g  Bajitian30  Tusizi15g  Yinyanghuo15g  Huangqin30g  Huangqi20g  Qinghao9g  Shudi15g  Sangjisheng15g  Gancao5g | N/A | Fuzi: anti-invasion and metastas  Baijitian, Tusizi：regulates IL-6 and TNF-α  Yinyanghuo, Huangqin, Huangqi, Sangjisheng:Pro-apoptotic, anti-invasion and metastas.  Qinghao:Target angiogenin and VEGF, pro-apoptotic.  Shudi: tumor microenvironment regulation  Gancao: Immune regulatory. | N/A | | N/A | | N/A | | N/A | | tid, 8 w | | Oral | | Granule | | | |
|  | Wang, et al 2022 | Huangqin Decoction | Huangqin 18 g  Baishao18 g  Gancao 10 g  Dazao 6g | N/A | Huangqin：Pro-apoptotic, anti-invasion and metastas, pro-necrosis.  Baishao：Pro-apoptotic, Immune regulatory, anti-inflammatory.Gancao:antibacterial, anti-inflammatory, hypoglycemic, lipid-lowering, antioxidant,  Dazao: antibacterial | N/A | | N/A | | N/A | | N/A | | bid, 200 ml, 8 w | | Oral | | Add 2,000 mL of water to decoct the traditional Chinese medicine compound to 400 mL of the remaining decoction.. | | | |
|  | Wang 2022 | Huangqin Decoction | Huangqin 18 g  Baishao18 g  Gancao 10 g  Dazao 6g | N/A | Huangqin：Pro-apoptotic, anti-invasion and metastas, pro-necrosis.  Baishao：Pro-apoptotic, Immune regulatory, anti-inflammatory.Gancao:antibacterial, anti-inflammatory, hypoglycemic, lipid-lowering, antioxidant,  Dazao: antibacterial | N/A | | N/A | | N/A | | N/A | | bid, 8 w | | Oral | | Add 2,000 mL of water to decoct the traditional Chinese medicine compound to 400 mL of the remaining decoction. | | | |

**Abbreviations:** TCM, traditional Chinese medicine; N/A, not applicable; w, weeks; qd, once daily; bid, twice daily.tid, third daily
