# Supplementary material for: Evidence and possible mechanism of Scutellaria baicalensis and its bioactive compounds for hepatocellular carcinoma treatment
Source: Ann Med. 2024 Jan 17;55(2):2247004. doi: 10.1080/07853890.2023.2247004 (PMC10795786; doi:10.1080/07853890.2023.2247004)
Supplement: Supplemental Material [file IANN_A_2247004_SM6826.zip › Table_S7.docx]

| **Table S7. The characteristics of cell experiments in vitro** | | | | |  |
| --- | --- | --- | --- | --- | --- |
| **Author, year** | **Cell Type** | **Cell origin (species)** | **Cell Tracking Method** | **Outcomes** | **Pathway** |
| Chiu et al, 2011 | HaCaT | Human HCC SK-Hep1  cells | MTT(cell viability)  FCM (apoptosis and cell cycle)  Boyden chamber(adhesion)  Scratch assays([migration](javascript:;))  IMC(NF-κB)  WB (NF-κB)  Zymography(MMP-2 and MMP-9) | Baicalein down-regulates MMP-9 and MMP-2 for the suppressive effects on invasion. | NF-κB pathway |
| Cui, 2008 | HaCaT | Human HCC HepG2 cells | MTT(cell viability)  IMC(Caspase-3, Bcl-2, Bax, P53)  WB(Caspase-3, Bcl-2, Bax, P53)  FCM (apoptosis and cell cycle) | BAI inhibits cell apoptosis by blocking the apoptosis mechanism of baicalin in S phase, which is related to the decrease of Bcl-2/Bax ratio and the activation of caspase3 cascade. | PI3K/AKT/mTOR  pathway |
| Hong et al, 2011 | HaCaT | Human HCC HepG2 cells | MTT(cell viability)  FCM (apoptosis and cell cycle)  PCR (CyclinD1)  IMC(CyclinD1, GSK3, ERK1/2, IKKα) | WOG induces HCC cells cycle arrest and suppresses tumor proliferation |  |
| Liang et al, 2012 | HaCaT | Human HCC  HepG2 | MTT(cell viability)  FCM (Apoptosis and cell cycle, MMP∆ψm)  WB(caspase-3,caspase9,p-MEK1,ERK1/2 ,Bad,and β-actin)  IHC(MAPK/ERK) | A decrease of MMP ψm and released of cytochrome c from mitochondria, and the following activation of caspase-9 and caspase-3,suggesting a mitochondrial signaling-related apoptosis was induced by BAE in HCC cells.  Bad is one of the downstream targets of BAE-induced inhibition of ERK | MEK-ERK  pathway |
| Xu et al, 2013 | HaCaT | Human HCC  HepG2, SMMC-7721, Hep3B | MTT(cell viability)  FCM (Apoptosis and cell cycle, Ca^2+^ and H_2_O_2_)  WB(UPR and PI3K/AKT-related protein) | WOG suppressed the viability of HCC cells through inducing apoptosis and necrosis.  WOG was able to activate the UPR and AKT pathway in HCC cells. | UPR and PI3K/AKT pathway |
| Zheng et al, 2014 | HaCaT | Human HCC H22, HepG2 | MTT(cell viability)  FCM (Apoptosis and cell cycle)  WB(cyclin D1, GSK-3β)  PCR( myc, Jun, cyclin D1)  IHC(AKT, cyclin D1) | BAE might regulate cyclin D1 transcription via a β-catenin-dependent mechanism, leading to cell cycle arrest at G0/G1 phase and impaired cancer cell proliferation. |  |
| Cen et al, 2020 | CSC | Human hepatocellular carcinoma cells | PCR (decoy receptor 3)  WB (decoy receptor 3)  CCK-8(proliferation)  FCM (cell cycle)  TW(apoptosis and migration) | BAE inhibited the expression of decoy receptor 3 in liver CSC cells , and affected the proliferation, cell cycle, apoptosis and migration. | N/A |
| Chang et al,2002 | HaCaT | Human hepatoblastoma G2 | SRB(Sulforhodamine B)  FCM (cell cycle)  WB(bcl-2) | BAE affected the cell apoptosis via inhibited the expression of bcl-2, and obtained the profile of DNA content. | N/A |
| Guo et al, 2007 | HaCaT | Human HCC BEL-7402 | Boyden chamber(adhesion)  FCM(cell cycle and apoptosis, integrinβ1， and E-cd) | BAI exerts an anti-adhesion effect on HCC cells, which may be related to inhibiting the expression of adhesion molecule E-Cd, promoting integrinβ expression, changing cell cycle and increasing cell apoptosis. | N/A |
| Guo et al, 2012 | HaCaT | Human HCC SMMC-7721 | PCR (Cx26, Cx43)  WB (Cx26)  IHC(Cx43) | Restoration or enhancement of gap junction intercellular communication induced by up-regulation of Cx26 and Cx43 is likely to be an important molecular mechanism by which baicalin inhibits tumor growth. | N/A |
| Guo et al, 2018 | HaCaT | Human HCC Bel-7402、Bel-7402/5- fu、Bel-7402/ADM | PCR (miR-181a-5p, notch2, Hes1, c-Myc, cyclin A2, and CDK2) FCM (cell cycle) | BAE could regulate the cell growth and reverse the chemotherapy resistance in HCC via modulating the expression of miR-181a-5p and the activity of notch2-c-Myc-cyclin A2/CDK2 signaling. | Hes1/c-Myc/cyclin A2/CDK2 |
| Han et al, 2014 | HaCaT | Human HCC BEL-7402 | FCM(cell cycle and apoptosis)  WB(CD24)  PCR(CD24)  CCK-8(proliferation) | BAE suppresses cell growth and cell survival through down-regulation of CD24 and c-myc. | N/A |
| Kuo et al, 2009 | HaCaT | Human hepatoma J5 cells | FCM (cell cycle,cell viability, caspase-3, Ca2+ and mitochondrial membrane potential level)  PCR (STAT3, CEPBD) WB (p53, p27, p21, Chk2, Wee1, Cdc25c, cyclin B1, Cdc2, Bax, Bcl-2, cytochrome c, Apaf-1, pro-caspase-9, AIF, Endo G, caspase-3, GRP78, GADD153, calalase, and Mn-SOD) FCM (Apoptosis and cell cycle) | The cytotoxicity of BAE in J5 cells is attributable to apoptosis mainly involving G2/M-arrest in an ER-dependent manner, via a mitochondria-dependent caspase pathway and as well as contributions of AIF and Endo G pathways. | AIF and  Endo G pathway |
| Li et al, 2015 | HaCaT | Human HCC BEL-7402 | FCM (cell cycle)  MTT(cell viability)  WB(Bcl‑2, Bax and p53) | WOG may act as an effective drug with anti-proliferative and apoptotic activity in HCC cells. Down-regulation of the Bcl-2/Bax signaling pathway in wogonoside-induced apoptosis. | Bcl-2/Bax pathway |
| Liu et al, 2016 | HaCaT | Human HCC  HepG2、BEL-7402 | CCK-8(proliferation)  PCR(CyclinD1,MMP-2,CyclinE,Bcl-2,CDK4,CDK6)  WB(ERK,AKT,EGFR,CyclinD1,MMP-2,CyclinE,Bcl-2,CDK4,CDK6,Caspase-9,caspase-3)  TW(apoptosis and migration)  FCM (Apoptosis and cell cycle) | WOG exerts its anticancer effects  through the inhibition of EGFR activity.  WOG promoted apoptosis, which was in parallel with the down-regulation of Bcl-2 protein and the cleavage of caspase-3  and caspase-9, and inhibited NF-κB  Signaling. | ERK/AKT  pathway |
| Matsuzaki et al, 1996 | HaCaT | Human HCC  Huh-7 | FCM(Fas) | The Fas-negative KIM-1 cells were sensitive to BAE-induced cell apoptosis, which does not induce the cell apoptosis by the Fas antigen. | N/A |
| Park et al, 1998 | HaCaT | Human HCC  ICIC-7 | MTT(cell viability)  PCR(QR) | BAI increased the level of QRmRNA in cultured HCC cells, indicating that baicalin-induced elevation of QR activity is mediated by enhanced QR gene expression. | TLR7/8 pathway |
| Park et al, 2014 | HaCaT | Human HCC  HepG2 | MTT(cell viability)  TW(apoptosis and migration)  GZ(MMP-2)  PCR(FOXM1、MMP-2)  WB(FOXM1、MMP-2) | Huangqin decreased metastasis through  inhibition of FOXM1 and these results correlated with MMP-2 inhibition. | N/A |
| Tan et al, 2015 | HaCaT | Human HCC MHCC97L | FCM ( CD11b,Ly6C, CD115, F4/80, CD86, CD206, RelA、IKKα、RelB、TRAF2、TRAF3 I )  PCR(TNF-α,IL12, IL-10,Arg1) | the tumour suppres-sive effect of baicalin was mediated by re-education of T AMs away from M2-like, towards tumour inhibiting M1-like pheno-type. This effect was regulated by activation of RelB/p52 pathway via TRAF2 lysosomal degradation-dependent pathway. BAI-induced autophagy was responsible for lysosomal degradation of TRAF2 as well as T AM repolarisation. | N/A |
| Tan et al, 2021 | HaCaT | Human HCC Hepa 1-6 | MTT(cell viability)  WB( PD-L1, LOXL4)  PCR( PD-L1, LOXL4) | BAI inhibited PD-L1 expression in LOXL4-treated macrophages  BAI intervention inhibited suppressive activity of LOXL4-treated macrophage on the proliferation and activation of cocultured T cells. | N/A |
| Wang et al, 2006 | HaCaT | Human HCC SMMC-7721 | FCM (cell cycle)  MTT(cell viability)  WB(Bcl‑2, Bax)  PCR(Bcl‑2, Bax) | WOG induces apoptosis in human hepatoma cell line SMMC7721 via the modulation of bcl-2/bax protein expression. | N/A |
| Wang et al, 2014 | HaCaT | Human HCC SMMC-7721, BEL-7402 | CCK-8(proliferation)  FCM (Ca2+, apoptosis and cell cycle)  WB(UPR, JNK, Bcl-2, Bcl-xL, Mcl-1) | BAE induces ER stress in HCC cells and activates UPR.  BAE inhibited the expression of LC-3I, LC-3II Atg5 and Beclin 1, which represents an important event during activation of autophagy. | N/A |
| Wang et al, 2015 | HaCaT | Human HCC  HepG2 | MTT(cell viability)  WB(LC3 , AKT, ULK1, mTOR)  FCM (apoptosis and cell cycle) | BAE triggered autophagy and inhibited the AKT/mTOR pathway in HepG2 cells. | AKT/mTOR  pathway |
| Wu et al, 2022 | HaCaT | Human HCC SMMC-7721 | CCK-8(proliferation)  FCM (apoptosis and cell cycle)  WB(YAP, TAZ, MOB1, LATS1) | WOG induced HCC cell cycle arrest and apoptosis probably by activating MOB1-LATS1 signaling to inhibit the activation of YAP and TAZ. | Hippo  pathway |
| Xiao et al, 2012 | HaCaT | Human HCC SMMC-7721 | TW(apoptosis and migration)  PCR(Ezrin, MMP-9, VEGF)  IHC(Ezrin, MMP-9, VEGF) | BAI inhibited Ezrin, mmp-9, VEGF mRNA and protein expression in tumor cells. | N/A |
| Yu et al, 2007 | HaCaT | Human HCC  BEL-7402 | MTT(cell viability)  WB(ROS, MMP, Caspase-9)  ELISA(Cytochrome c) | Induction of apoptosis on Bel-7402 cells by WOG could be associated with ROS production, GSH depletion and mitochondrial dysfunction. | N/A |
| Yu et al, 2016 | HaCaT | Human HCC  HepG2, SMMC7221 | MTT(cell viability)  TUNEL(cell apoptosis)  PCR(IRE1, ATF6, PERK, S1P, S2P)  WB(IRE1, ATF6, PERK, S1P, S2P) | BAI induces cell apoptosis by activating ATF6 signaling pathway in endoplasmic reticulum (ER) stress.  S2P, rather than S1P is the molecular target for BAI in inducing ER stress-mediated HCC cell apoptosis. | ATF6  pathway |
| Zhang et al, 2012 | HaCaT | Human HCC SMMC-7721 | MTT(cell viability)  IHC(Βeclin 1)  WB(Beclin 1, CD147) | Autophagy induced by BAI involves down-regulation of CD147 in SMMC-7721 cells in vitro. | N/A |
| Zhao et al, 2007 | HaCaT | Human HCC  HepG2 | MTT(cell viability)  PCR(survivin) | BAE can induce the apoptosis of HCC cell line HepG2and the probable mechanism is to depress the expression of anti apoptosis gene survivin. | N/A |
| Zhong et al,2013 | HaCaT | Human HCC  HepG2 | MTT(cell viability)  FCM (apoptosis and cell cycle)  WB(HDAC1, HDAC2) | Baicalein can inhibit the growth of HepG2 cells in a dose-dependent manner. | N/A |
| Li, 2007 | HaCaT | Human HCC  BEL-7402 | MTT(cell viability)  WB(bcl-2, bax, P53) | BAI inhibits cell apoptosis by blocking the apoptosis mechanism of baicalin in S phase, which is related to the decrease of Bcl-2/Bax ratio and the activation of caspase3 cascade |  |

**Abbrevations**: CSC, Cancer Stem Cell; DC, Dendritic Cells; PBMCs, Peripheral blood mononuclear cell; HUVECS, Human Umbilical Vein Endothelial Cells; BAE, Baicalein; BAI, Baicalin; WOG, Wogonin; IHC, immunohistochemistry; ELISA,enzyme-linked immuno sorbent assay; FCM, Flow Cytometry; WB, Western Bolt; PCR, Polymerase Chain Reaction; CCK-8, Cell counting kit-8; TW, Transwell Assay; GZ,Gelatin zymography; IL, interleukin; VEGF, vascular endothelial growth factor; LC:Light Chain
