# Supplementary figures and images for: Evidence and possible mechanism of Scutellaria baicalensis and its bioactive compounds for hepatocellular carcinoma treatment
Source: Ann Med. 2024 Jan 17;55(2):2247004. doi: 10.1080/07853890.2023.2247004 (PMC10795786; doi:10.1080/07853890.2023.2247004)

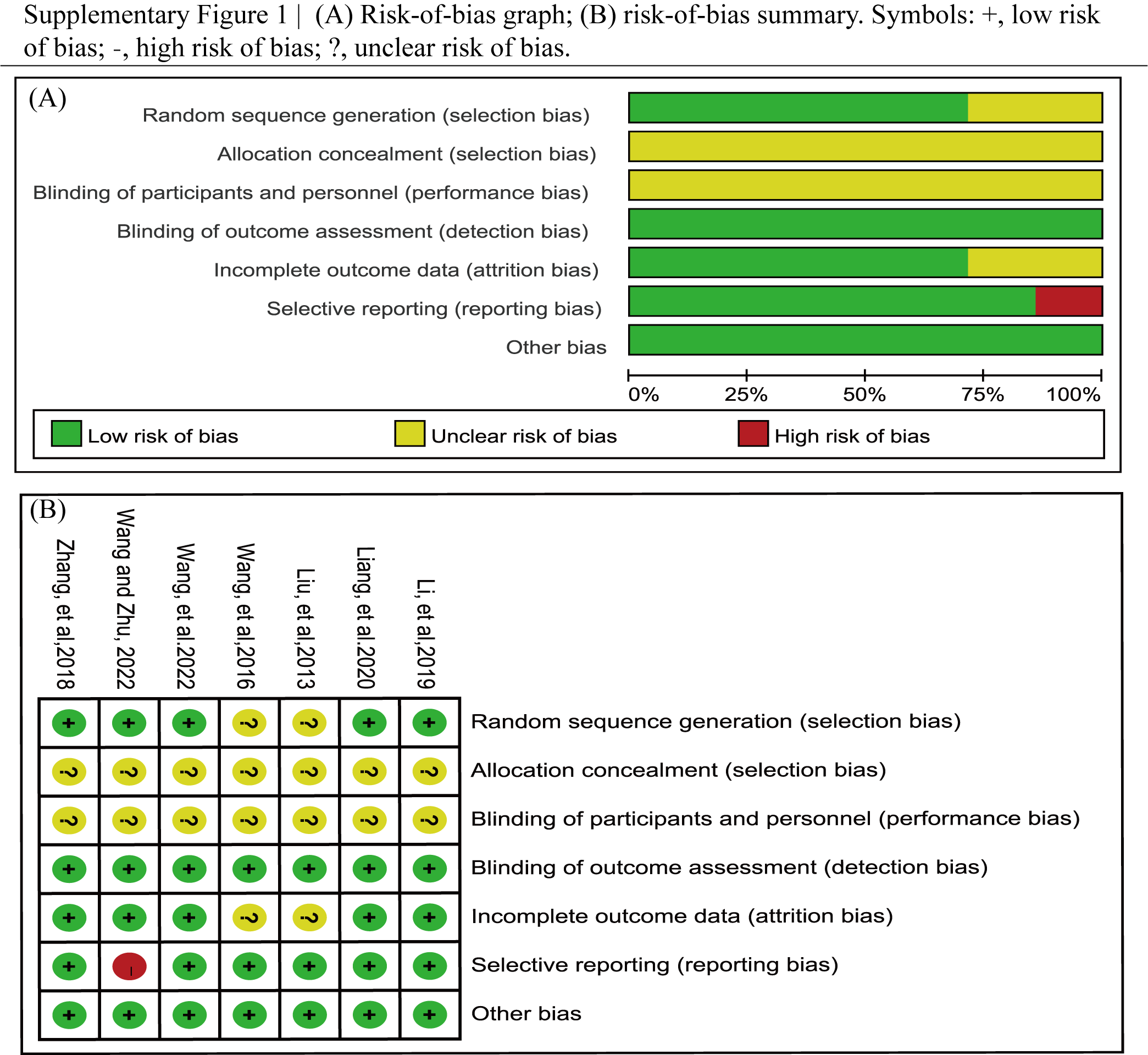

Supplement: Supplemental Material [file IANN_A_2247004_SM6826.zip › Figure_S1.tif]

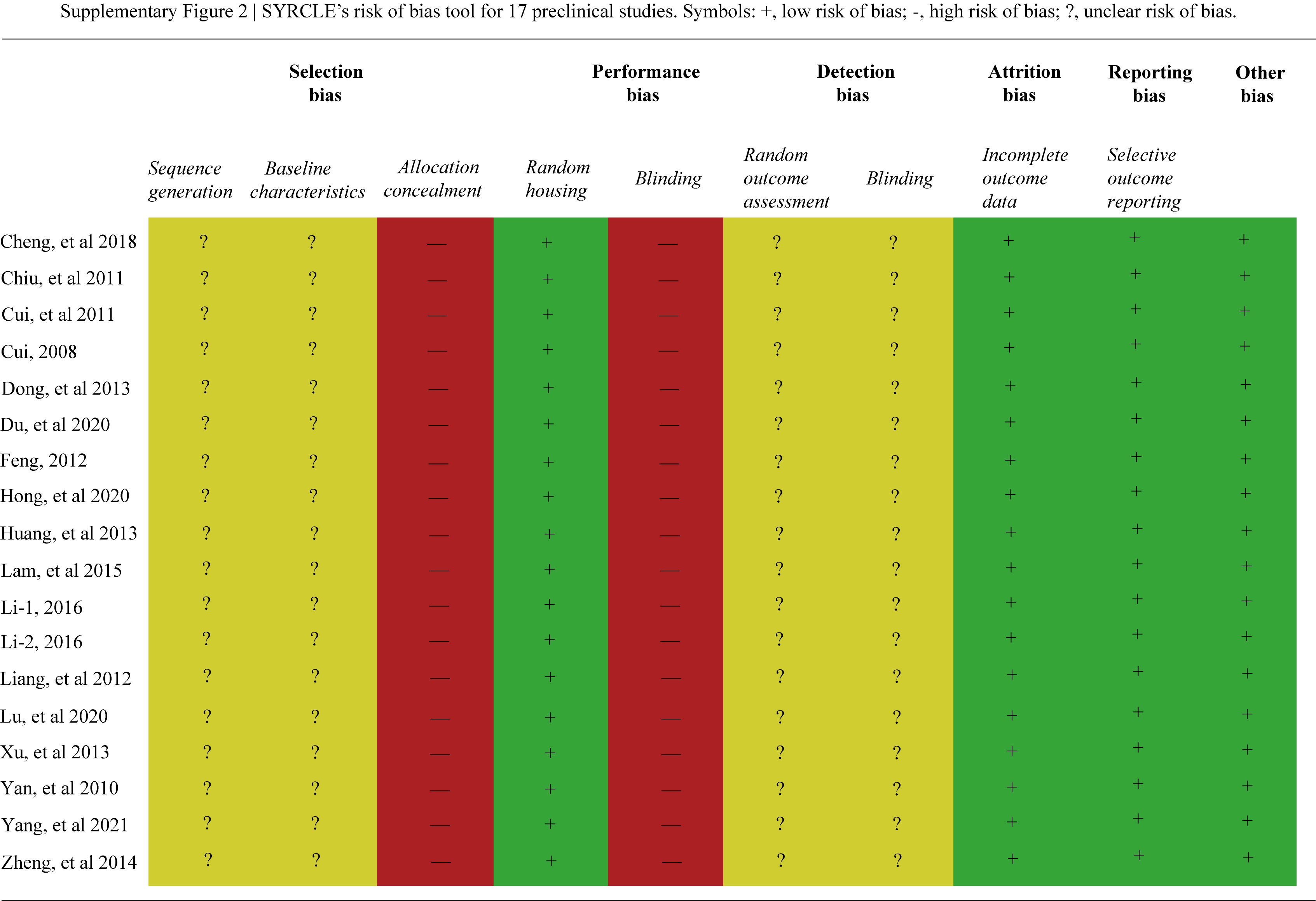

Supplement: Supplemental Material [file IANN_A_2247004_SM6826.zip › Figure_S2.tif]
