# Supplementary material for: Evidence and possible mechanism of Scutellaria baicalensis and its bioactive compounds for hepatocellular carcinoma treatment
Source: Ann Med. 2024 Jan 17;55(2):2247004. doi: 10.1080/07853890.2023.2247004 (PMC10795786; doi:10.1080/07853890.2023.2247004)
Supplement: Supplemental Material [file IANN_A_2247004_SM6826.zip › Table_S4.doc]

| Study ID | Species | Age/Gender/Weight | Type of tumor transplantation | Drug | Control | Administration | Time | Outcomes | Number of group | Dose |
| --- | --- | --- | --- | --- | --- | --- | --- | --- | --- | --- |
| Cheng, 2018 | Kunming species | 6-8week/18-22g/female | H22 | Huangqin | NS | i.p | 6d | 1, | / | 0.2ml/d |
| Chiu, 2011 | BALB/c-nu | 4-6week/18-20g/female | SK-Hep1 | Baicalein | NS | S.C | 5d | 1 | L;M;H | 5,10,20mg/Kg/d |
| Cui, 2012 | Kunming species | 8week/18-22g/half male and half female | H22 | Baicalein | NS;CTX | i.g | 15d | 1,6,8 | L;M;H | 20,40,80mg/Kg/d |
| Cui, 2008 | Kunming species | 18-22g/male | S180 | Baicalin | NS，CTX | i.g | 10d | 1,5,9 | L;M;H | 0.125mg,0.25mg,0.5mg/Kg/d |
| Dong, 2013 | BALB/c-nu | 4week/18-23g/half male and half female | SMMC-7721 | Baicalin | NS | i.p | 16d | 1,3,4,6,7,8 | / | 100mg/kg/od |
| Du, 2015 | BALB/c-nu | 4-6week/14-18g/male | HepG2 | Baicalein | NS | i.p | 30d | 6,9 | L;M;H | 15,50,100mg/Kg/d |
| Feng, 2012 | BALB/c-nu | 3week/male | SMMC-7721 | Baicalein | NS | i.p | 14d | 1 |  |  |
| Hong, 2020 | BALB/c-nu | 5week/male | MHCC97L | Wogonin | NS,sorafenib | i.p | 35d | 2 | L;H | 30,50mg/Kg/d |
| Huang, 2013 | Kunming species | 6-8week/18-22g/half male and half female | H22 | Wogonin | NS;CTX | i.p | 10d | 1 | L;M;H | 25,50,100μg/mL |
| Li, 2016 | ICR | 18-22g/male | S180 | Huangqin | NS;5-FU | i.g | 10d | 1.2.3.5. | L;M;H | 0.9,1.8,2.7/Kg/d |
| Li, 2016 | ICR | 18-22g/male | H22 | Huangqin | NS;5-FU | i.g | 10d | 1.2.3.5.9 | L;M;H | 0.9,1.8,2.7/Kg/d |
| LU, 2020 | Kunming species | 6-8week/18-22g/half male and half female | H22 | Baicalin | NS;CTX | i.g | 15d | 1,2,5 | L;M;H | 50,100,200mg/Kg/d |
| Lam, 2015 | BALB/c-nu | 6week/female | HepG2 | Huangqin | NS,sorafenib | i.g | 7d | 8 | / | 1.0g/Kg/d |
| Xu, 2013 | BALB/c-nu | 6-8week/18-22g/male | SMMC-7721 | Wogonin | NS;5-FU | i.v | 21d | 1,2 | L;M;H | 15,30,60mg/Kg/od |
| Yan, 2010 | SDrat | 180-210g/male | DEN | Huangqin | NS | i.g | 140d | 2,3,5 | / | 4.3g/Kg/d |
| Yang, 2021 | C57BL/6 | 8week/female | Hepa 1-6 | Huangqin | NS,sorafenib | i.g | 7d | 2,8 | / | 1.0/Kg/d |
| Zheng, 2014 | ICR | 18-22g/male | H22 | Baicalein | NS;CTX | i.g | 12d | 1,2 | L;H | 50mg,100mg/Kg/d |
| Liang, 2012 | BALB/c-nu | 4week/male | HepG2 | Baicalein | NS | i.g | 21d | 1 | / | 20 mg/kg/d |

Supplementary Table 2 | The characteristics of the 17 preclinical studies

**Abbreviations：**i.p, intraperitoneally injection; s.c, subcutaneous injection; i.g,intragastric administration; NS, Nacl; CTX, cytoxan
