# Supplementary material for: Evidence and possible mechanism of Scutellaria baicalensis and its bioactive compounds for hepatocellular carcinoma treatment
Source: Ann Med. 2024 Jan 17;55(2):2247004. doi: 10.1080/07853890.2023.2247004 (PMC10795786; doi:10.1080/07853890.2023.2247004)
Supplement: Supplemental Material [file IANN_A_2247004_SM6826.zip › Table_S5_clean.doc]

**Table S5** Meta-analysis of preclinical outcome in vivo studies

|  | **Experimental** | | | **Control** | | | **Std. Mean Difference** |  |
| --- | --- | --- | --- | --- | --- | --- | --- | --- |
| **Study or Subgroup** | **Mean** | **SD** | **Total** | **Mean** | **SD** | **Total** | **IV. FiXed. 95% Cl** | **P value** |
| **Weight of tumor** |  |  |  |  |  |  |  |  |
| **Baicalein** |  |  |  |  |  |  |  |  |
| Cui, 2012-H | 0.61 | 0.18 | 10 | 1.59 | 0.28 | 10 | -3.99 [-5.62, -2.35] | _ |
| Cui, 2012-M | 0.88 | 0.24 | 10 | 1.59 | 0.28 | 10 | -2.61 [-3.87, -1.35] | _ |
| Cui, 2012-L | 1.03 | 0.37 | 10 | 1.59 | 0.28 | 10 | -1.63 [-2.68, -2.59] | _ |
| Chiu, 2011-M | 0.3 | 0.09 | 8 | 1.25 | 0.05 | 8 | -12.34 [-17.36, -7.32] | _ |
| Chiu, 2011-H | 0.28 | 0.1 | 8 | 1.25 | 0.05 | 8 | -11.60 [-16.33, -6.87] | _ |
| Chiu, 2011-L | 0.53 | 0.11 | 8 | 1.25 | 0.05 | 8 | -7.97 [-11.29, -4.64] | _ |
| Zheng,2014-H | 0.21 | 0.06 | 6 | 0.98 | 0.15 | 6 | -6.22 [-9.46, -2.98] | _ |
| Zheng, 2014-L | 0.4 | 0.07 | 6 | 0.98 | 0.15 | 6 | -4.57 [-7.08, -2.07] | _ |
| Feng, 2012 | 0.77 | 0.88 | 10 | 1.01 | 0.15 | 10 | -0.36 [-1.25, 0.52] | _ |
| Subtotal (95% Cl)  I^2^ = 89% |  |  |  |  |  |  | -2.25 [-2.77, -1.72] | P < 0.00001 |
| **Baicalin** |  | | |  |  |  |  |  |
| Cui, 2008-H | 0.61 | 0.18 | 12 | 1.59 | 0.28 | 12 | -4.02 [-5.50, -2.54] | _ |
| Cui, 2008-M | 0.88 | 0.24 | 12 | 1.59 | 0.28 | 12 | -2.63 [-3.77, -1.49] | _ |
| Cui, 2008-L | 1.03 | 0.37 | 12 | 1.59 | 0.28 | 12 | -1.65 [-2.60, -0.70] | _ |
| Dong, 2013 | 0.5 | 0.03 | 10 | 0.73 | 0.04 | 10 | -6.23 [-8.56, -3.90] | _ |
| Lu, 2020-H | 1.91 | 1.47 | 10 | 5.5 | 3.34 | 10 | -1.33 [-2.32, -0.34] | _ |
| Lu, 2020-M | 2.08 | 1.54 | 10 | 5.5 | 3.34 | 10 | -1.26 [-2.24, -0.28] | _ |
| Lu, 2020-L | 2.75 | 2.93 | 10 | 5.5 | 3.34 | 10 | -0.84 [-1.76, 0.08] | _ |
| Subtotal (95% Cl)  I^2^ = 81% |  |  |  |  |  |  | -1.82 [-2.24, -1.41] | P< 0.00001 |
| **Huangqin** |  | | |  |  |  |  |  |
| Cheng, 2018 | 0.64 | 0.32 | 10 | 1.17 | 0.36 | 10 | -1.49 [-2.51, -0.47] | _ |
| Li-2, 2016-M | 1.03 | 0.46 | 12 | 1.7 | 0.56 | 12 | -1.26 [-2.15, -0.37] | _ |
| Li-1, 2016-M | 1.13 | 0.64 | 10 | 1.77 | 0.41 | 10 | -1.14 [-2.10, -0.18] | _ |
| Li-2, 2016-L | 1.35 | 0.39 | 12 | 1.7 | 0.56 | 12 | -0.70 [-1.53, 0.13] | _ |
| Li-2, 2016-H | 1.39 | 0.31 | 12 | 1.7 | 0.56 | 12 | -0.66 [-1.49, 0.16] | _ |
| Li-1, 2016-H | 1.48 | 0.63 | 10 | 1.77 | 0.41 | 10 | -0.52 [-1.42, 0.37] | _ |
| Li-1, 2016-L | 1.48 | 0.66 | 10 | 1.77 | 0.41 | 10 | -0.51 [-1.40, 0.39] | _ |
| Subtotal (95% Cl)  I^2^ = 0% |  |  |  |  |  |  | -0.86 [-1.20, -0.53] | P< 0.00001 |
| **Wogonin** |  | | |  |  |  |  |  |
| Xu, 2013-H | 0.52 | 0.18 | 6 | 1.64 | 0.61 | 6 | -2.30 [-3.89, -0.71] | _ |
| Xu, 2013-M | 0.82 | 0.33 | 6 | 1.64 | 0.61 | 6 | -1.54 [-2.90, -0.18] | _ |
| Xu, 2013-L | 1.25 | 0.25 | 6 | 1.64 | 0.61 | 6 | -0.77 [-1.97, 0.42] | _ |
| Subtotal (95% Cl)  I^2^ = 14% |  |  |  |  |  |  | -1.39 [-2.18, -0.61] | P=0.0005 |
| Total (95% Cl)  I^2^ = 82% |  | | |  |  |  | -2.14 [-2.70, -1.58] | P< 0.00001 |
| **Apoptosis of tumor** |  | | |  |  |  |  |  |
| **Baicalein** |  |  |  |  |  |  |  |  |
| Cui, 2012-L | 15.3 | 6.7 | 10 | 2.9 | 0.3 | 10 | 2.50 [1.27, 3.74] | _ |
| Cui, 2012-M | 24.6 | 3.1 | 10 | 2.9 | 0.3 | 10 | 2.61 [6.06, 12.82] | _ |
| Cui, 2012-H | 39.1 | 4.9 | 10 | 2.9 | 0.3 | 10 | 1.63 [6.42, 13.55] | _ |
| Du, 2020-L | 15.26 | 3.87 | 10 | 8.79 | 2.35 | 10 | 12.34 [0.83, 3.04] | _ |
| Du, 2020-M | 35.78 | 4.45 | 10 | 8.79 | 2.35 | 10 | 11.60 [4.60, 9.93] | _ |
| Du, 2020-H | 54.38 | 5.09 | 10 | 8.79 | 2.35 | 10 | 11.01 [7.11, 14.92] | _ |
| Subtotal (95% Cl)  I^2^ = 91% |  |  |  |  |  |  | 3.56 [2.83, 4.30] | P < 0.00001 |
| **Baicalin** |  |  |  |  |  |  |  |  |
| Dong, 2013 | 13.6 | 1.1 | 10 | 2.2 | 1.9 | 10 | 7.03 [4.45, 9.62] | _ |
| Subtotal (95% Cl) |  |  |  |  |  |  | 7.03 [4.45, 9.62] | P< 0.00001 |
| Total (95% Cl)  I^2^ = 90% |  |  |  |  |  |  | 6.68 [4.07, 9.29] | P< 0.00001 |
| **Body weight** |  |  |  |  |  |  |  |  |
| **Baicalein** |  |  |  |  |  |  |  |  |
| Zheng, 2014-L | 30.6 | 1.3 | 6 | 31.33 | 2.08 | 6 | -0.73 [-2.69, 1.23] | _ |
| Zheng, 2014-H | 30.51 | 1.33 | 6 | 31.33 | 2.08 | 6 | -0.82 [-2.80, 1.16] | _ |
| Subtotal (95% Cl)  I^2^ = 0% |  |  |  |  |  |  | -0.77 [-2.17, 0.62] | P = 0.28 |
| **Baicalin** |  |  |  |  |  |  |  |  |
| LU, 2020-L | 34.41 | 5.69 | 10 | 35.76 | 3.96 | 10 | -1.35 [-5.65, 2.95] | _ |
| LU, 2020-M | 34.45 | 5.1 | 10 | 35.76 | 3.96 | 10 | -1.31 [-5.31, 2.69] | _ |
| LU, 2020-H | 36.27 | 6.7 | 10 | 35.76 | 3.96 | 10 | 0.51 [-4.31,5.33] | _ |
| Subtotal (95% Cl)  I^2^ = 0% |  |  |  |  |  |  | -0.83 [-3.34, 1.67] | P=0.51 |
| **Huangqin** |  |  |  |  |  |  |  |  |
| Li-1, 2016-L | 31.2 | 3.9 | 10 | 31.9 | 2.4 | 10 | -0.70 [-3.54, 2.14] | _ |
| Li-1, 2016-M | 30.1 | 2.9 | 10 | 31.9 | 2.4 | 10 | -1.80 [-4.13, 0.53] | _ |
| Li-1, 2016-H | 30.7 | 2.5 | 10 | 31.9 | 2.4 | 10 | -1.20 [-3.35, 0.95] | _ |
| Li-2, 2016-L | 34.1 | 4.2 | 12 | 33.9 | 2.5 | 12 | 0.20 [-2.57, 2.97] | _ |
| Li-2, 2016-M | 33.8 | 2.7 | 12 | 33.9 | 2.5 | 12 | -0.10 [-2.18, 1.98] | _ |
| Li-2, 2016-H | 34.6 | 2.0 | 12 | 33.9 | 2.5 | 12 | 0.70 [-1.11, 2.51] | _ |
| Yan, 2010 | 396.41 | 61.3 | 23 | 357.57 | 74.84 | 18 | 38.84 [-3.86, 81.54] | _ |
| Yang, 2021 | 89.68 | 3.88 | 5 | 92.78 | 7.33 | 5 | -3.10 [-10.37, 4.17] |  |
| Subtotal (95% Cl)  I^2^ = 6% |  |  |  |  |  |  | -0.41 [-1.32, 0.50] | P=0.37 |
| **Wogonin** |  |  |  |  |  |  |  |  |
| Hong, 2020-1 | 21.32 | 1.4 | 6 | 23.1 | 1.14 | 6 | -1.78 [-3.22, -0.34] | _ |
| Hong, 2020-2 | 23.74 | 1.28 | 6 | 23.1 | 1.14 | 6 | 0.64 [-0.73, 2.01] | _ |
| Xu, 2013-1 | 23.6 | 2.92 | 6 | 25.45 | 2.07 | 6 | -1.85 [-4.71, 1.01] | _ |
| Xu, 2013-2 | 24.09 | 2.21 | 6 | 25.45 | 2.07 | 6 | -1.36 [-3.78, 1.06] | _ |
| Xu, 2013-3 | 25.08 | 2.07 | 6 | 25.45 | 2.07 | 6 | -0.37 [-2.71, 1.97] | _ |
| Subtotal (95% Cl)  I^2^ = 41% |  |  |  |  |  |  | -1.41 [-2.26, -0.57] | P=0.10 |
| total (95% Cl)  I^2^ =0% |  |  |  |  |  |  | -0.61 [-1.16, -0.07] | P=0.03 |
| **Liver index** |  |  |  |  |  |  |  |  |
| **Baicalein** |  |  |  |  |  |  |  |  |
| Zheng, 2014-L | 7.41 | 0.39 | 6 | 7.56 | 0.19 | 6 | -0.45 [-1.60, 0.70] | _ |
| Zheng, 2014-H | 7.16 | 0.41 | 6 | 7.56 | 0.19 | 6 | -1.16 [-2.42, 0.11] | _ |
| Subtotal (95% Cl)  I^2^ = 0% |  |  |  |  |  |  | -0.77 [-1.62,0.08] | P = 0.08 |
| **Baicalin** |  |  |  |  |  |  |  |  |
| Dong, 2013 | 2.98 | 0.41 | 10 | 2.51 | 0.35 | 10 | 1.18 [0.21,2.15] | _ |
| Subtotal (95% Cl) |  |  |  |  |  |  | 1.18 [0.21,2.15] | P=0.02 |
| **Huangqin** |  |  |  |  |  |  |  |  |
| Li-1, 2016-L | 8.11 | 2.04 | 10 | 8.84 | 0.83 | 10 | -0.45 [-1.34, 0.44] | _ |
| Li-1, 2016-M | 8.31 | 1.32 | 10 | 8.84 | 0.83 | 10 | -0.46 [-1.35, 0.43] | _ |
| Li-1, 2016-H | 8.2 | 0.74 | 10 | 8.84 | 0.83 | 10 | -0.78 [-1.70, 0.14] | _ |
| Li-2, 2016-L | 6.4 | 1.39 | 12 | 6.48 | 0.45 | 12 | -0.07 [-0.88, 0.73] | _ |
| Li-2, 2016-M | 6.76 | 0.72 | 12 | 6.48 | 0.45 | 12 | 0.45 [-0.36, 1.26] | _ |
| Li-2, 2016-H | 6.42 | 0.36 | 12 | 6.48 | 0.45 | 12 | -0.14 [-0.94, 0.66] | _ |
| Yan, 2010 | 6.71 | 0.51 | 8 | 8.84 | 0.53 | 6 | -3.85 [-5.83, -1.86] | _ |
| Subtotal (95% Cl)  I^2^ = 65% |  |  |  |  |  |  | -0.31 [-0.66, 0.03] | P=0.07 |
| total (95% Cl)  I^2^ =68% |  |  |  |  |  |  | -2.14 [-2.70, -1.58] | P=0.14 |
| **Spleen index** |  |  |  |  |  |  |  |  |
| **Baicalein** |  |  |  |  |  |  |  |  |
| Cui, 2012-L | 5.45 | 0.37 | 10 | 5.12 | 0.28 | 10 | 0.96 [0.03, 1.90] |  |
| Cui, 2012-M | 8.04 | 0.34 | 10 | 5.12 | 0.28 | 10 | 8.98 [5.75,12.21] |  |
| Cui, 2012-H | 8.53 | 0.53 | 10 | 5.12 | 0.28 | 10 | 7.71 [4.90,10.51] |  |
| Zheng, 2014-L | 0.85 | 0.07 | 6 | 0.84 | 0.09 | 6 | 0.11 [-1.02, 1.25] |  |
| Zheng, 2014-H | 0.94 | 0.01 | 6 | 0.84 | 0.09 | 6 | 1.44 [0.11,2.77] |  |
| Subtotal (95% Cl)  I^2^ = 91% |  |  |  |  |  |  | 1.42[0.81, 2.03] | P=0.004 |
| **Baicalin** |  |  |  |  |  |  |  |  |
| Lu, 2020-L | 9.96 | 4.36 | 10 | 12.07 | 4.03 | 10 | -0.48 [-1.37, 0.41] |  |
| Lu, 2020-M | 6.88 | 2.68 | 10 | 12.07 | 4.03 | 10 | -1.45 [-2.46, -0.44] |  |
| Lu, 2020-H | 6.61 | 3.04 | 10 | 12.07 | 4.03 | 10 | -1.46 [-2.48, -0.45] |  |
| Subtotal (95% Cl)  I^2^ = 29% |  |  |  |  |  |  | -1.08 [-1.63, -0.52] | P=0.0002 |
| **Huangqin** |  |  |  |  |  |  |  |  |
| Li-1, 2016-L | 0.85 | 0.35 | 10 | 0.77 | 0.17 | 10 | 0.28 [-0.60, 1.16] |  |
| Li-1, 2016-M | 0.85 | 0.23 | 10 | 0.77 | 0.17 | 10 | 0.38 [-0.51, 1.27] |  |
| Li-1, 2016-H | 0.79 | 0.15 | 10 | 0.77 | 0.17 | 10 | 0.12 [-0.76, 1.00] |  |
| Li-2, 2016-L | 0.54 | 0.15 | 12 | 0.63 | 0.11 | 12 | -0.66 [-1.49, 0.17] |  |
| Li-2, 2016-M | 0.54 | 0.1 | 12 | 0.63 | 0.11 | 12 | -0.83 [-1.67, 0.01] |  |
| Li-2, 2016-H | 0.58 | 0.11 | 12 | 0.63 | 0.11 | 12 | -0.44 [-1.25, 0.37] |  |
| Yan, 2010 | 0.38 | 0.09 | 12 | 0.48 | 0.1 | 12 | -1.01 [-1.87, -0.16] |  |
| Subtotal (95% Cl)  I^2^ = 39% |  |  |  |  |  |  | -0.33 [-0.65, 0.01] | P=0.04 |
| total (95% Cl)  I^2^ =85% |  |  |  |  |  |  | -0.18 [-0.43,0.07] | P=0.16 |
| **Caspase-3** |  |  |  |  |  |  |  |  |
| **Baicalein** |  |  |  |  |  |  |  |  |
| Cui, 2012-L | 142.54 | 13.36 | 10 | 100.67 | 4.45 | 10 | 4.03 [2.38, 5.67] | _ |
| Cui, 2012-M | 197.77 | 16.93 | 10 | 100.67 | 4.45 | 10 | 7.51 [4.77, 10.26] | _ |
| Cui, 2012-H | 280.62 | 28.51 | 10 | 100.67 | 4.45 | 10 | 8.45 [5.40, 11.50] |  |
| Subtotal (95% Cl)  I^2^ = 77% |  |  |  |  |  |  | 6.45 [3.50, 9.40] | P< 0.0001 |
| **Baicalin** |  |  |  |  |  |  |  |  |
| Dong, 2013 | 31.31 | 6.09 | 10 | 20.08 | 5.01 | 10 | 1.93 [0.83, 3.03] | _ |
| Subtotal (95% Cl) |  |  |  |  |  |  | 1.93 [0.83, 3.03] | P=0.00006 |
| **Huangqin** |  |  |  |  |  |  |  |  |
| Lam, 2015 | 8.32 | 3.39 | 5 | 0.68 | 0.54 | 5 | 2.84 [0.82, 4.87] | _ |
| Yang, 2021 | 6.28 | 3.64 | 5 | 1.26 | 0.53 | 5 | 1.74 [0.16, 3.32] | _ |
| Subtotal (95% Cl)  I^2^ = 0% |  |  |  |  |  |  | -0.31 [-0.66, 0.03] | P=0.00007 |
| Total (95% Cl)  I^2^ =83% |  |  |  |  |  |  | -2.14 [-2.70, -1.58] | P< 0.0001 |
| **VEGF** |  |  |  |  |  |  |  |  |
| **Baicalein** |  |  |  |  |  |  |  |  |
| Du, 2020-L | 38.79 | 4.13 | 10 | 53.85 | 5.15 | 10 | -3.09 [-4.47, -1.71] |  |
| Du, 2020-M | 23.84 | 3.37 | 10 | 53.85 | 5.15 | 10 | -6.60 [-9.05, -4.16] |  |
| Du, 2020-H | 8.55 | 4.13 | 10 | 53.85 | 5.15 | 10 | -9.29 [-12.63, -5.96] |  |
| Subtotal (95% Cl)  I^2^ = 86% |  |  |  |  |  |  | -4.56 [-5.69, -3.43] | P< 0.0001 |
| **Huangqin** |  |  |  |  |  |  |  |  |
| Li-2, 2016-L | 91 | 4 | 12 | 100 | 0.01 | 12 | -3.07 [-4.31, -1.83] |  |
| Li-2, 2016-M | 89 | 14 | 12 | 100 | 0.01 | 12 | -1.07 [-1.94, -0.21] |  |
| Li-2, 2016-H | 47 | 9 | 12 | 100 | 0.01 | 12 | -8.04 [-10.65, -5.43] |  |
| Subtotal (95% Cl)  I^2^ = 93% |  |  |  |  |  |  | -2.16 [-2.85, -1.48] | P＜0.0001 |
| Total (95% Cl)  I^2^ =91% |  |  |  |  |  |  | -2.81 [-3.39, -2.22] | P＜0.0001 |
