# Supplementary material for: Evidence and possible mechanism of Scutellaria baicalensis and its bioactive compounds for hepatocellular carcinoma treatment
Source: Ann Med. 2024 Jan 17;55(2):2247004. doi: 10.1080/07853890.2023.2247004 (PMC10795786; doi:10.1080/07853890.2023.2247004)
Supplement: Supplemental Material [file IANN_A_2247004_SM6826.zip › Table_S6_clean.doc]

**Table S6** Meta-analysis for Bcl-2, MMP-2, MMP-9, Caspase-3, and CyclinD1 (in vitro studies)

|  | **Experimental** | | | **Control** | | | **Std. Mean Difference** |  |
| --- | --- | --- | --- | --- | --- | --- | --- | --- |
| **Study or Subgroup** | **Mean** | **SD** | **Total** | **Mean** | **SD** | **Total** | **IV. Fixed. 95% Cl** | **P value** |
| **Bcl-2** |  |  |  |  |  |  |  |  |
| **Baicalein** |  |  |  |  |  |  |  |  |
| Cui, 2008 | 32.21 | 3.35 | 3 | 59.26 | 4.45 | 3 | -5.70 [-11.43, 0.03] | _ |
| Subtotal (95% Cl) |  |  |  |  |  |  | -5.70 [-11.43, 0.03] | P=0.05 |
| **Wogonin** |  | | |  |  |  |  |  |
| Liu, 2016-H | 0.32 | 0.02 | 3 | 0.99 | 0.03 | 3 | -21.02 [-41.39, -0.66] | _ |
| Liu, 2016-L | 0.88 | 0.02 | 3 | 0.99 | 0.03 | 3 | -3.45 [-7.15, 0.25] | _ |
| Liu, 2016-M | 0.67 | 0.01 | 3 | 0.99 | 0.03 | 3 | -11.45 [-22.62, -0.28] | _ |
| Subtotal (95% Cl)  I^2^ = 54% |  |  |  |  |  |  | -4.73 [-8.18, -1.27] | P=0.007 |
| Total (95% Cl)  I^2^ =32% |  |  |  |  |  |  | -4.99 [-7.95, -2.02] | P=0.001 |
| **MMP-2** |  |  |  |  |  |  |  |  |
| **Baicalein** |  |  |  |  |  |  |  |  |
| Chiu, 2011 | 0.58 | 0.25 | 3 | 0.99 | 0.12 | 3 | -1.67 [-3.95, 0.60] | _ |
| Subtotal (95% Cl) |  |  |  |  |  |  | -1.67 [-3.95, 0.60] | P=0.15 |
| **Huangqin** |  |  |  |  |  |  |  |  |
| Park, 2014-H | 0.13 | 0.11 | 3 | 0.79 | 0.01 | 3 | -6.76 [-13.48, -0.04] | _ |
| Park, 2014-L | 0.42 | 0.13 | 3 | 0.79 | 0.01 | 3 | -3.21 [-6.70, 0.28] | _ |
| Subtotal (95% Cl)  I^2^ = 0% |  |  |  |  |  |  | -3.96 [-7.06, -0.87] | P=0.01 |
| **Wogonin** |  |  |  |  |  |  |  |  |
| Liu, 2016-H | 0.08 | 0.01 | 3 | 0.99 | 0.01 | 3 | -72.80 [-143.11, -2.49] | _ |
| Liu, 2016-L | 0.38 | 0.01 | 3 | 0.99 | 0.01 | 3 | -48.80 [-95.95, -1.65] | _ |
| Liu, 2016-M | 0.34 | 0.02 | 3 | 0.99 | 0.01 | 3 | -32.89 [-64.68, -1.09] | _ |
| Subtotal (95% Cl)  I^2^ = 0% |  |  |  |  |  |  | -42.17 [-66.85, -17.48] | P=0.0008 |
| Total (95% Cl)  I^2^ =62% |  |  |  |  |  |  | -2.69 [-4.52, -0.87] | P=0.004 |
| **MMP-9** |  |  |  |  |  |  |  |  |
| **Baicalein** |  |  |  |  |  |  |  |  |
| Chiu, 2011 | 0.37 | 0.13 | 3 | 0.99 | 0.12 | 3 | -3.96 [-8.11, 0.18] | _ |
| Subtotal (95% Cl) |  |  |  |  |  |  | -3.96 [-8.11, 0.18] | P=0.06 |
| **Baicalin** |  |  |  |  |  |  |  |  |
| Xiao, 2012-H | 21.85 | 3.41 | 3 | 41.48 | 3.55 | 3 | -4.51 [-9.15, 0.13] | _ |
| Xiao, 2012-L | 33.26 | 4.07 | 3 | 41.48 | 3.55 | 3 | -1.72 [-4.03, 0.59] | _ |
| Subtotal (95% Cl)  I^2^ = 10% |  |  |  |  |  |  | -2.27 [-4.34, -0.21] | P=0.03 |
| Total (95% Cl)  I^2^ =0% |  |  |  |  |  |  | -2.61 [-4.46, -0.76] | P=0.006 |
| **Caspase-3** |  |  |  |  |  |  |  |  |
| **Baicalein** |  |  |  |  |  |  |  |  |
| Kuo, 2009 | 0.79 | 0.05 | 3 | 0.19 | 0.03 | 3 | 0.60 [0.53, 0.67] | _ |
| Liang, 2012-H | 3.55 | 0.19 | 3 | 0.99 | 0.01 | 3 | 2.56 [2.34, 2.78] | _ |
| Liang, 2012-L | 1.5 | 0.42 | 3 | 0.99 | 0.01 | 3 | 0.51 [0.03, 0.99] | _ |
| Liang, 2012-M | 2.91 | 0.52 | 3 | 0.99 | 0.01 | 3 | 1.92 [1.33, 2.51] | _ |
| Total (95% Cl)  I^2^ =99% |  |  |  |  |  |  | 0.78 [0.71, 0.84] | P< 0.00001 |
| **CyclinD1** |  |  |  |  |  |  |  |  |
| **Baicalein** |  |  |  |  |  |  |  |  |
| Zheng, 2014-H | 0.33 | 0.07 | 3 | 1 | 0.01 | 3 | -0.67 [-0.75, -0.59] |  |
| Zheng, 2014-L | 0.21 | 0.1 | 3 | 1 | 0.01 | 3 | -0.79 [-0.90, -0.68] |  |
| Subtotal (95% Cl)  I^2^ =65% |  |  |  |  |  |  | -0.72 [-0.84, -0.61] | P< 0.00001 |
| **Wogonin** |  |  |  |  |  |  |  |  |
| Hong, 2020-H | 3.99 | 0.64 | 3 | 1.01 | 0.13 | 3 | 2.98 [2.24, 3.72] | _ |
| Hong, 2020-L | 2.3 | 0.4 | 3 | 1.01 | 0.13 | 3 | 1.29 [0.81, 1.77] | _ |
| Liu, 2016-H | 0.28 | 0.01 | 3 | 1 | 0.01 | 3 | -0.72 [-0.74, -0.70] | _ |
| Liu, 2016-L | 0.47 | 0.02 | 3 | 1 | 0.01 | 3 | -0.53 [-0.56, -0.50] | _ |
| Liu, 2016-M | 0.67 | 0.01 | 3 | 1 | 0.01 | 3 | -0.33 [-0.35, -0.31] | _ |
| Subtotal (95% Cl)  I^2^ =100% |  |  |  |  |  |  | -0.02 [-0.27, 0.22] | P=0.84 |
| Total (95% Cl)  I^2^ =100% |  |  |  |  |  |  | -0.27 [-0.46, -0.07] | P=0.007 |
